# Porous Phenazine‐bridged Tetraoxa[8]Circulenes for Selective Gold Recovery and Heterogeneous Catalysis

**DOI:** 10.1002/anie.6586260

**Published:** 2026-04-08

**Authors:** Patrick W. Fritz, Eylül Attar, Timur Ashirov, Krzysztof Piech, Ali Coskun

**Affiliations:** ^1^ Department of Chemistry University of Fribourg Fribourg Switzerland

**Keywords:** gold capture, heterogeneous catalysis, polymeric tetraoxa[8]circulenes, porous polymers, TOCs

## Abstract

The sustainable recovery of precious metals is critical for securing strategic resources while reducing the environmental footprint of mining. Among these, the selective recovery of gold from electronic waste represents a particularly attractive yet challenging target. Herein, we report the synthesis of phenazine‐based porous tetraoxa[8]circulene, PpTOC, incorporating heterocyclic crown ether moieties for efficient gold capture. The PpTOC combines a high surface area over 1200 m^2^ g^−^^1^ with aza‐crown ether‐like cavities and high heteroatom content, enabling high gold uptake capacities of up to 860 mg g^−^^1^ under acidic conditions. Importantly, the polymer demonstrates selective recovery of gold from electronic waste even in the presence of high concentrations of competing metal ions such as copper. The recovered material, containing a mixture of Au(III), Au(I), and Au(0) species, has been directly employed as a heterogeneous catalyst in a sequential alkylation–annulation reaction. Critically, switching from aprotic toluene to protic ethanol redirects the reaction outcome from alkylation to annulation, enabling divergent product selectivity using the same heterogeneous catalyst simply by changing the solvent.

## Graphical abstract

Gold recycling from electronic waste using porous materials is an emerging concept to reduce the environmental impact of gold mining. In this work, we report the synthesis of a highly porous phenazine‐derived tetraoxa[8]circulene polymer and its utilization in gold capture and heterogeneous catalysis.

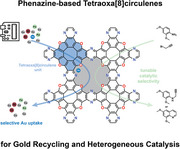

## 1 Introduction

Precious metals such as gold, palladium, and platinum play important roles in society, in engineering, catalysis, and energy applications [1, 2]. The use of these metals is integral, particularly in catalysis and electronics applications, and suitable replacements are hard to find [3, 4]. However, the sourcing of precious metals results in an immense environmental impact due to CO_2_ emissions associated with the mining and processing of the raw materials [5], the highly toxic waste produced upon enrichment and purification [6, 7], and the human cost due to often poor work environments. As a result, environmental groups and governments have pushed towards more efficient use and recycling of precious metals. Over the past decades, huge stockpiles of electronic waste have been amassed. However, environmentally friendly means to recycle precious metals from waste electronics remain scarce. Printed circuit boards (PCBs), for example, contain a mix of metals, including tin, copper, nickel, and gold, the recycling of which would be highly beneficial in our efforts to reduce emissions from mining and refining of new resources.

Recent developments in porous materials, including metal–organic frameworks (MOFs) [8], covalent organic frameworks (COFs) [9, 10, 11], and porous organic polymers (POPs) [12, 13, 14, 15], have focused on developing materials that can withstand the extreme conditions of waste streams containing gold ions while maintaining high gold capacities. An integral part of most of these materials is a reductive mechanism promoted by redox‐active linkers that enable the conversion of Au(III) to Au(0). Among previously reported systems, phenazine‐containing polymers [14, 15] have repeatedly been used as they facilitate the complexation of the Au(III) ions in aqueous solutions and reduction due to their redox‐active properties. The use of captured gold in these systems as heterogeneous catalysts, however, is quite rare and focused on single‐step transformations such as the carboxylation of allylic systems [16, 17]. The use of recycled metals as heterogeneous catalysts represents a sustainable approach to catalysis by decreasing the environmental footprint of such systems. Heterogeneous catalysts offer significant advantages over their homogeneous counterparts, including recyclability, enhanced stability, and compatibility with continuous‐flow processes, rendering them particularly attractive for large‐scale and sustainable chemical manufacturing. However, their application has traditionally focused on single‐step organic transformations, where control over activity and selectivity can be achieved by optimizing the reaction conditions. In contrast, the extension of heterogeneous catalysts to multi‐step organic transformations remains far less explored. Achieving such pathway control with a single heterogeneous catalyst represents a significant advance, as it brings the level of reactivity tuning typically associated with homogeneous systems into robust, recyclable, and scalable catalytic platforms. In this direction, sequential alkylation‐annulation reactions [18], which are typically carried out using homogeneous gold catalysts, represent attractive targets for heterogeneous alternatives. Annulation reactions provide access to heterocycles such as quinolines, which are important structural motifs in many pharmaceutical compounds [19]. Therefore, the synthesis of these compounds using gold recovered from e‐waste is a sustainable and industrially relevant approach.

**SCHEME 1:**
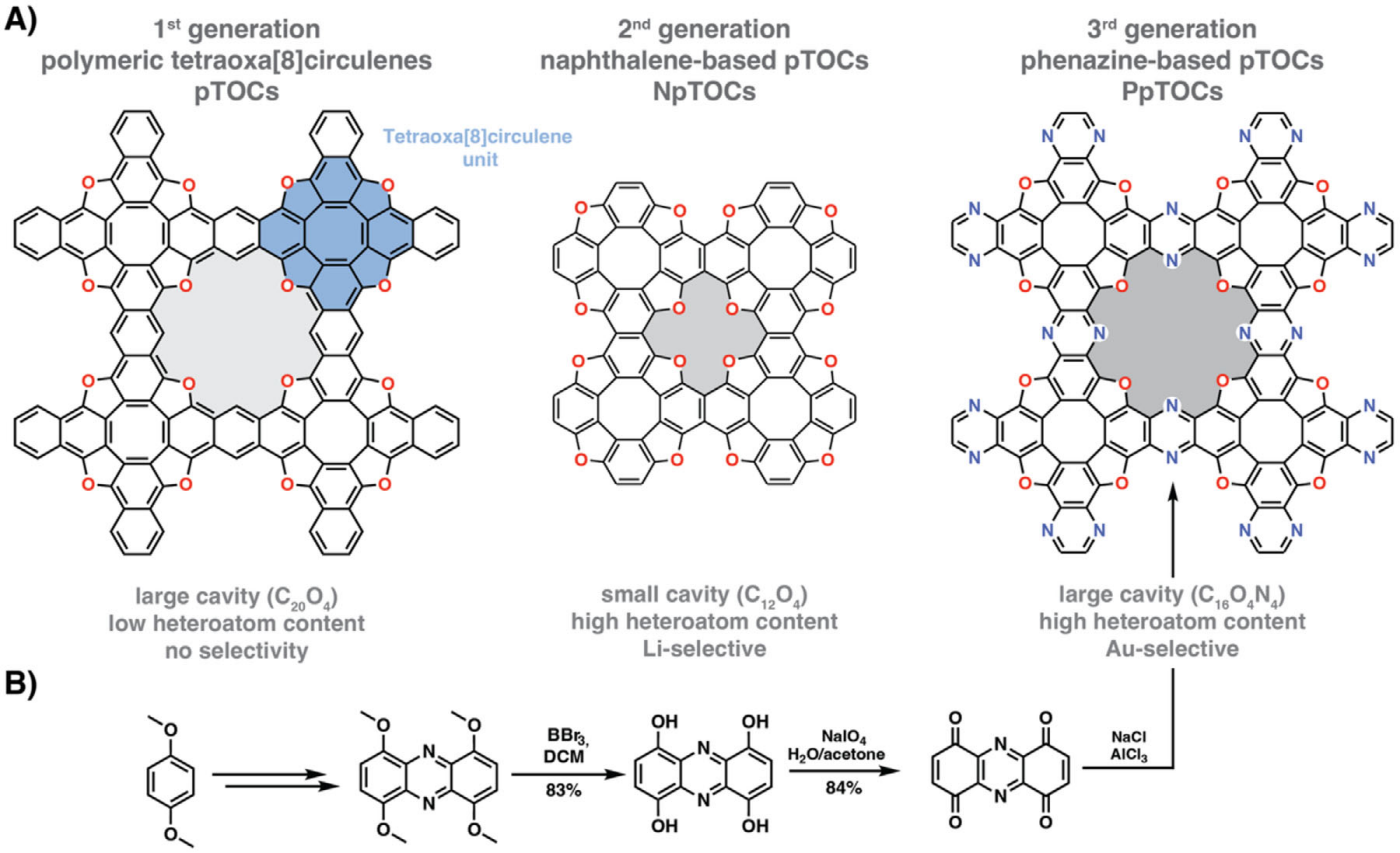
A) Polymeric tetraoxa[8]circulene‐based porous organic polymers and their features. A tetraoxa[8]circulene unit is highlighted in blue. The different pore environments created by using different precursors from the first to the third generation of pTOCs are highlighted in grey. B) Schematic illustration of the synthetic procedure for the synthesis of PpTOCs starting from 1,4‐dimethoxybenzene.

Polymeric tetraoxa[8]circulenes (pTOCs) have been previously synthesized and employed in our group as organic semiconductors [20] and for lithium recycling [21] due to their conjugated backbones and high chemical and physical stability. While the first pTOCs were not suitable for ion capture applications due to their large pore aperture and low heteroatom content, NpTOCs (naphthalene‐based pTOCs) had much smaller pores mimicking heterocyclic crown ethers, enabling selective Li‐ion capture. We hypothesized that a phenazine‐derived pTOC would improve the ion capture capabilities of pTOCs by increasing their heteroatom content and by providing an aza‐crown ether‐like coordination environment while maintaining its large pore aperture. In this direction, herein, we report (Scheme 1A) the synthesis and characterization of phenazine‐derived pTOCs (PpTOCs) and their use as a sorbent for gold recovery from acidic media, even in competitive environments. Moreover, the recovered material, Au@PpTOC, containing Au(III), Au(I), and Au(0), has been used as a heterogeneous catalyst in the sequential alkylation–annulation reactions. The use of toluene as a nonpolar, aprotic solvent enables the selective formation of the alkylation product. In contrast, switching to polar protic ethanol promotes annulation reaction, leading to the formation of the corresponding quinoline derivative. This result demonstrates that divergent product selectivity can be achieved with the same heterogeneous catalyst, featuring recycled gold, simply by changing the solvent, enabling a two‐step synthesis of quinolines without any modification of the catalyst composition.

## 2 Results and Discussion

**FIGURE 1:**
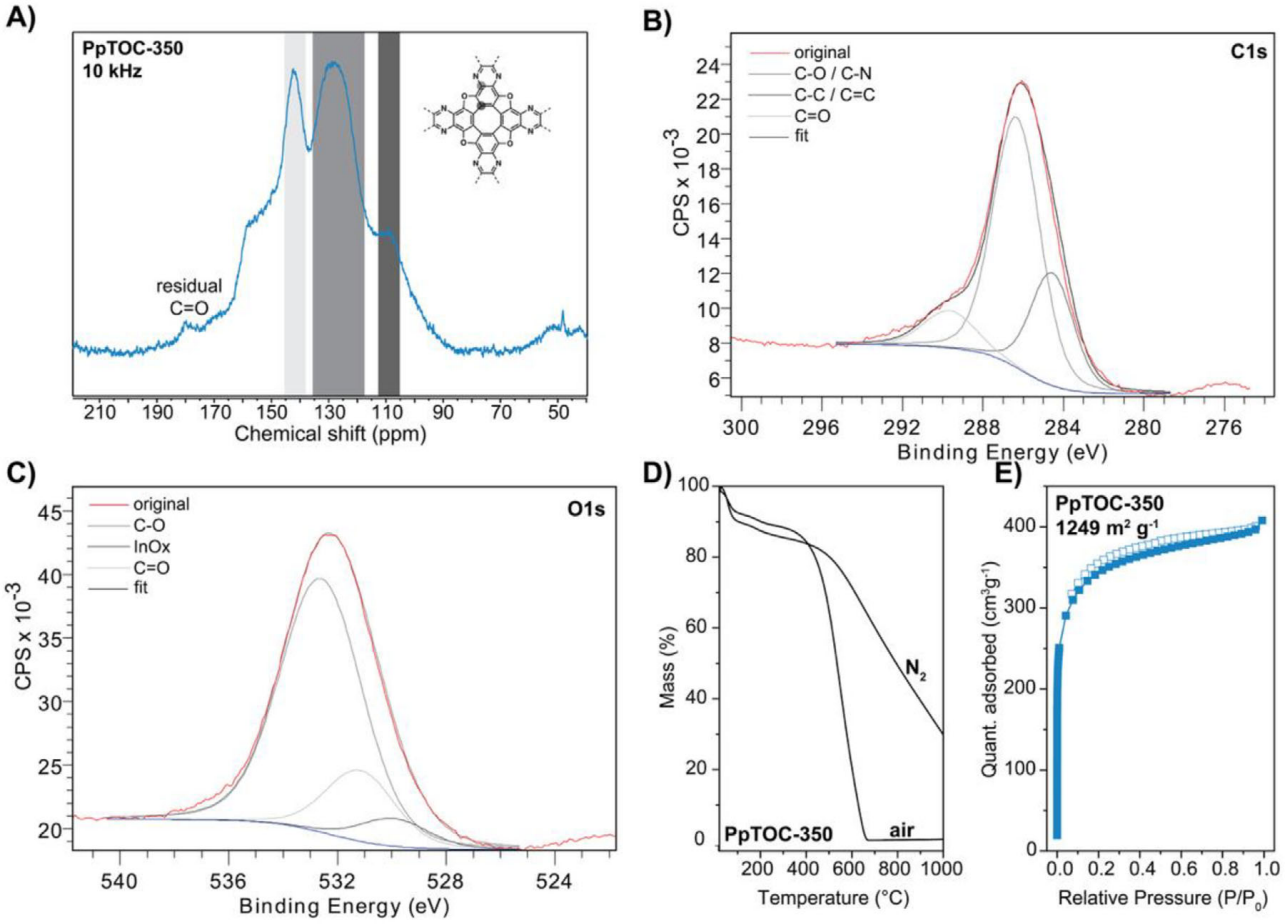
Characterization of PpTOC‐350. A) CP‐MAS ^13^C NMR spectrum of PpTOC‐350. Residual carbonyls are highlighted. B) C 1s, and C) O 1s deconvolutions of the HR‐XPS narrow scans of PpTOC‐350. *Note*: The samples were supported on an indium foil for the measurement. Thus, signals related to indium and its oxides (denoted as InOx) are present in survey spectra. D) TGA of PpTOC‐350 under nitrogen and air. E) BET surface area analysis of PpTOC‐350.

In our previous work on polymeric TOCs, we showed that pTOCs could be prepared under ionothermal and solvothermal conditions in the presence of Lewis (e.g., AlCl_3_) or Brønsted (e.g., MsOH) acids as reaction mediators. We reasoned that, similar to pTOCs, which were prepared from 1,4,5,8‐anthracene tetrone, phenazine‐based polymeric TOCs (PpTOCs) could be prepared from 1,4,5,8‐phenazine tetrone. A suitable precursor was prepared in a five‐step procedure starting from 1,4‐dimethoxybenzene and relied on a Wohl–Aue reaction, a subsequent ether deprotection, and a periodate‐mediated oxidation (Scheme 1B, see Supplementary Information for details). Moving forward, we attempted the synthesis of PpTOCs under ionothermal (previously used for the preparation of pTOCs) and solvothermal conditions (previously used for preparing NpTOCs). PpTOCs could not be obtained under solvothermal conditions using either methanesulfonic acid or triflic acid [22], likely due to protonation of the phenazine N‐moieties. However, PpTOCs could be obtained under ionothermal conditions using an aluminum chloride—sodium chloride eutectic salt mixture. PpTOCs were thus prepared under ionothermal conditions at 250°C, 300°C, and 350°C and were named PpTOC‐X, where X stands for the reaction temperature. The Fourier transform infrared (FTIR) spectra showed only minor differences between the reaction temperatures. The presence of C═N stretching modes at 1620 cm^−1^ confirms the presence of phenazine moieties, and C─O modes at 1150 and 840 cm^−1^ indicate the formation of the TOC backbone. However, PpTOC‐250 and PpTOC‐300 showed more pronounced carbonyl C═O stretching modes at 1680 cm^−1^ (Figure S1). Similarly, the cross‐polarization magic angle spinning ^13^C nuclear magnetic resonance (CP‐MAS ^13^C NMR) spectra showed more pronounced carbonyl signals around 180 ppm, indicating unreacted or terminal carbonyl moieties as well as reaction intermediates [22] (Figures 1A and S2). The presence of residual carbonyls is also evident in high‐resolution X‐ray photoelectron spectroscopy (HR‐XPS) spectra of PpTOC‐350. The C1s deconvolution of PpTOC‐350 showed the presence of residual carbonyls at 289.6 eV, a major contribution of C─O/C ═N at 286.3 eV, and sp^2^ carbons at 284.5 eV. Similarly, the O 1s spectrum could be deconvoluted into C─O species at 532.5 eV, carbonyl species at 530.8 eV, and surface oxygen originating from the support (Figure 1B,C). Lastly, the N 1s spectrum showed only a single nitrogen species at 399.1 eV, characteristic of phenazine‐type nitrogen moieties [14] (Figure S3). The thermal stability of the polymer was assessed using thermogravimetric analysis (TGA), which showed that PpTOC‐250 and ‐300 were stable up to 300°C under air, while PpTOC‐350 was stable up to 370°C (Figures 1D and S4). It should be noted that PpTOCs showed water uptake during the weighing process of the measurement of around 10 wt%. All polymers were fully decomposed around 600°C, indicating that a negligible amount of metal species (residual Al as Al_2_O_3_) was bound in the polymers’ pores. The amorphous character of the polymers was confirmed by powder X‐ray diffraction (PXRD) analysis (Figure S5).

The textural properties of the polymers were assessed using the Brunauer–Emmett–Teller (BET) theory applied to N_2_ isotherms obtained at 77 K. A clear increase in surface area with increasing reaction temperature is visible, resulting in BET surface areas of 148, 482, and 1249 m^2^ g^−1^ for PpTOC‐250, ‐300, and ‐350, respectively (Table 1, Figures 1E, S6A, and Figure S7). All polymers showed Type‐I isotherms, and their pore‐size distribution (PSD) was assessed using NLDFT kernels. The PSD of all polymers confirms their microporous nature, and PpTOC‐350 features prominent peaks at 0.91 nm and a secondary peak at 0.45 nm. Additionally, a broader feature between 1.2–2.5 nm indicates the disordered character of the polymer that results from random packing (Figure S6B). Considering the high heteroatom content of the polymers and their microporous nature, we investigated their CO_2_ uptake at different temperatures and measured relatively good uptake capacities of 1.47, 2.96, and 4.31 mmol g^−1^ at 273 K and 1.1 bar for PpTOC‐250, ‐300, and ‐350, respectively (Figure S8). While CO_2_ capture is still considered a high‐priority topic [9, 13], we hypothesized that the heterocyclic crown ethers in PpTOCs, in combination with their high chemical and thermal stability, would render them suitable for the recovery of precious metals such as gold owing to the presence of heteroatoms and ideal cavity size. Importantly, recovering precious metals from waste streams can significantly reduce the CO_2_ emissions associated with conventional mining practices.

**TABLE 1:** Textural properties and CO_2_ uptake capacities of PpTOCs.

							CO_2_ uptake (mmol g^−1^)		
Sample	BET^a^ (m^2^ g^−1^)	S_micro_ ^b^ (m^2^ g^−1^)	S_ext_ ^c^ (m^2^ g^−1^)	V_total_ ^d^ (cm^3^ g^−1^)	V_micro_ ^e^ (cm^3^ g^−1^)	V_ext_ ^f^ (cm^3^ g^−1^)	273 K	298 K	323 K
PpTOC‐250	148.0	117.9	30.1	0.095	0.066	0.029	1.47	0.95	0.67
PpTOC‐300	482.5	396.7	85.7	0.247	0.161	0.086	2.96	1.94	1.33
PpTOC‐350	1249.0	914.2	334.8	0.630	0.383	0.247	4.31	2.74	1.89

^a^ BET surface areas were calculated over the valid pressure range as determined by the Rouquerol plots (see Supplementary Information).

^b^ Micropore surface area was calculated using the *t*‐plot method.

^c^ S_ext_ = S_total_ − S_micro_

^d^ Total pore volume was obtained at P/P_0_ = 0.99.

^e^ Micropore volume was calculated using the *t*‐plot method.

^f^ V_ext_ = V_total_ − V_micro_

**FIGURE 2:**
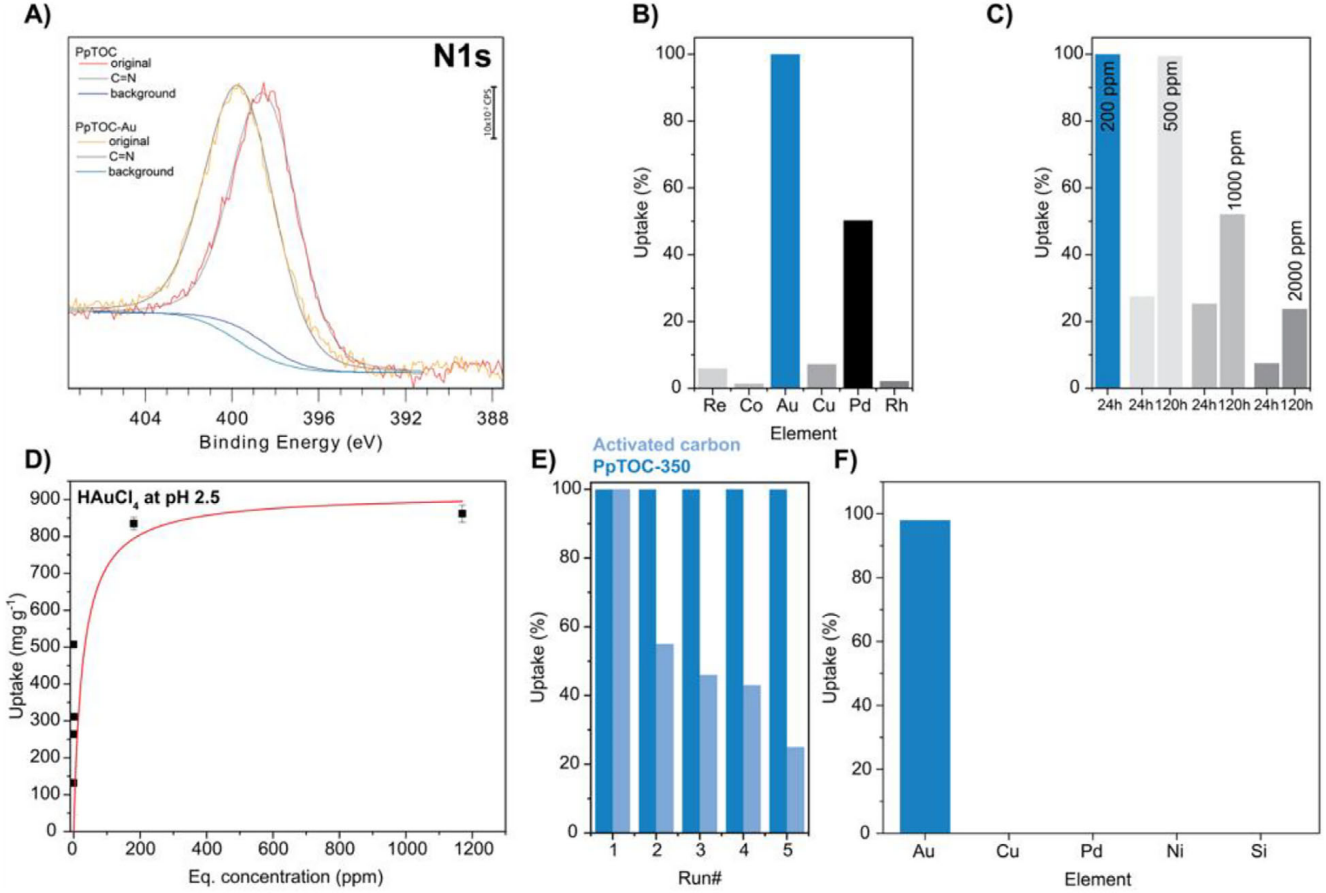
Gold uptake performance of PpTOC. A) Maximum uptake using HAuCl_4_ in an aqueous solution without adjusting the pH. B) Gold uptake using unadjusted Au standard solutions after 24 h and 5 days. C) Consecutive uptake experiments with recycling of PpTOC (dark blue) and activated carbon (light blue) using thiourea/HCl, showing a drop in uptake performance if activated carbon is used. D) Selectivity test in a mixed solution of Au with competitive ions. E) Au recycling test of extracted PCBs. F) Deconvolution of the N 1s narrow scan of PpTOC‐350 before and after Au uptake.

The recovery of gold from waste, particularly from electronic waste, has gained considerable interest in recent years, and several research groups have shown that gold can be recovered from aqueous solutions using phenazine‐containing polymers due to a reductive mechanism that converts Au(III) ions to elemental gold [14, 15]. To test our hypothesis, we exposed PpTOC‐350—chosen for its high surface area and low number of residual carbonyl groups—to a 200 ppm Au(III) solution as well as to a mixture of Au, Re, Co, Cu, Pd, and Rh (30 ppm each). In both cases, after 24 h, we could not detect any residual gold ions in the solutions using inductively coupled plasma optical‐emission spectroscopy analysis (ICP‐OES), confirming our initial hypothesis. PXRD of the polymer after filtration and drying showed the presence of elemental gold (Figure S11C), thus confirming that gold reduction takes place. X‐ray photoelectron spectroscopy (XPS) measurements of PpTOC‐350 after gold uptake further corroborated this (Figures S9 and S10). While the deconvoluted narrow scans showed little change in the composition of the polymer, a clear peak for Au4f was observed, the deconvolution of which revealed a mixture of Au(0), Au(I), and Au(III) (Figure S11B). After observing Au(0) with XPS measurements, we also conducted scanning electron microscopy with energy‐dispersive X‐ray spectroscopy (SEM‐EDX, Figure S12) analyses. The EDX images clearly show that gold nanoparticles were obtained after gold capture by PpTOC. Additionally, the N 1s peak shifted to higher energies, indicating interactions between the metal ions and the pore, thus highlighting the critical role of the crown ether‐like cavity and nitrogen atoms [14] (Figures 2A and S9–S11). The uptake experiment under competitive conditions showed that gold is taken up preferentially—due to the high affinity of the polymer to gold and its subsequent reduction that removes gold from the polymer—with only Pd competing at low concentrations, while only minor amounts of Re, Co, Cu, or Rh are taken up (Figure 2B).

Initial experiments to determine the maximum Au uptake of PpTOC‐350 focused on using stock solutions as received without altering the pH of the solutions. However, it was determined that the pH of a solution plays an important role when using phenazine‐based systems due to the protonation of the phenazines. Our experiments showed that while all the gold can be removed from a 200‐ppm stock solution in 24 h, much lower uptakes were observed for 500 and 1000 ppm solutions (Note: the pH of the 1000‐ppm standard solution is 0.1). By extending the experiment for several days, the Au uptake improved significantly, indicating the role pH (or, more precisely, the presence of protons) plays in the uptake performance (Figure 2C). For that reason, literature reports often showcase gold uptake at different pH values. To test this, pH adjustment experiments were performed with aqueous ammonia and dilute NaOH solutions (Figures S13 and S14). However, both solutions showed precipitation of gold from the solution at varying time intervals. Precipitation such as the one reported here is commonly observed when adjusting the pH of gold solutions—gold loss from solutions has also been observed during long‐term storage without pH adjustment [23]. However, this is often neglected in gold capture literature, which makes it difficult to determine the exact amount of Au that was converted/taken up by the polymer and how much was precipitated. Due to that, we used stock solutions prepared from HAuCl_4_·3H_2_O with double‐distilled water, which did not show any gold precipitation after prolonged storage. The pH of the solution was determined to be 2.5, resulting in a maximum uptake capacity of 860 mg g^−1^ (Figure 2D, Table S2 for comparison). Besides high uptake capacity, the uptake kinetics are also a key parameter when assessing the performance of sorption materials. Initial experiments using 500 ppb stock solutions, which are commonly used in literature, showed exceptionally fast uptake, resulting in near‐quantitative gold removal from the solutions in less than one minute (Figure S15A). To further investigate the kinetics, the experiment was repeated with a more concentrated Au solution (around 25 ppm). Although the uptake was slower, even under these conditions, near‐quantitative uptake was achieved in less than 3 h, thus demonstrating the high affinity of the PpTOC towards gold (Figure S15B and C). A gold capture experiment was also conducted under a halogen lamp and in the dark for comparison. As expected, the gold uptake capacity increased to 1256 mg g^−1^ owing to the reduction of gold ions under the halogen lamp exposure over 72 h. (Figure S16) In the dark, PpTOC captured gold until the cavity was saturated, preventing further uptake (948 mg g^−1^) in 72 h. SEM‐EDX analyses (Figure S17) showed the presence of gold nanoparticles after gold capture under a halogen lamp.

Activated carbon (AC) is often used to recover gold from cyanide solutions at high pH owing to its high surface area and low cost [24, 25]. Accordingly, we tested a commercially available activated carbon sample with a surface area of 833 m^2^ g^−1^ (Figure S18) under the same Au uptake conditions as employed for PpTOC. ICP‐OES analysis revealed complete uptake of gold for both PpTOC and AC. After the initial uptake experiment, we performed recycling experiments up to five cycles by treating the PpTOC and AC with a 1:1 solution of HCl and thiourea (see details of the recycling experiment in the Supporting Information) to remove the gold. While PpTOC's performance remained consistent, AC's performance decreased drastically owing to its low mechanical stability (Figure 2E). These results demonstrate the robust gold capture performance of PpTOC over multiple cycles. This result prompted us to investigate whether any chemical changes occurred in the polymer network. Figure S19 shows PXRD patterns of PpTOC‐350 after one and five uptake‐recycling cycles. The lack of reflections corresponding to Au nanoparticles (see Figure S11C) demonstrates the complete removal of gold. These findings were further corroborated by the HR‐XPS survey and narrow scans presented in Figures S20 and S21. The C, N, and O deconvolutions show that the material remained stable, while the Au4f regions showed that only traces of gold could be detected. We also noted that residual sulfur species remained in the material, likely caused by the residual thiourea. Moreover, FT‐IR and CP‐MAS ^13^C NMR spectra after recycling were found to be identical (Figure S22), indicating that the polymer backbone remains intact after recycling experiments. Lastly, we would also like to note that these recycling experiments show that after the removal of gold, the N 1s energies are shifted back to their original energies when compared to the N 1s measurements of Au@PpTOC. To test whether PpTOC could be used in real‐life gold recovery applications, trashed printed circuit boards (random access memory kits and graphic cards) were used as gold surrogates. Although cyanide leaching is well‐established and commonly used for both ores and some e‐waste processing [26], we used a more environmentally friendly method introduced by Yang and coworkers, using aqueous solution of *N*‐bromosuccinimide (NBS) and pyridine (Py) [16, 27]. Despite the presence and high concentration of Cu (over 350 ppm, 80 times more compared to Au, see Table S1), gold was selectively adsorbed using the PpTOC in 4 h (Figure 2F).

After observing that PpTOC selectively captures gold even under competitive conditions. We explored the use of recovered polymer, Au@PpTOC, as a heterogeneous catalyst in a sequential alkylation–annulation reaction. It has been demonstrated that homogeneous gold catalysts featuring Au (I) or Au (III) can be used in alkylation–annulation reactions [18], where both Au(I) and Au(III)‐based homogeneous catalysts are effective for the annulation reaction in the synthesis of quinolines [28], which represent a key building block in numerous pharmaceutical compounds. Furthermore, these examples also show that alkylation–annulation reactions can be achieved with different reaction conditions using either Au (I) or Au (III) homogeneous catalysts. Since the Au 4f XPS spectrum of Au@PpTOC shows the presence of Au (0), Au (I), and Au (III) states, it suggests that multiple oxidation states of Au can be confined within the polymeric framework. Accordingly, we comparatively evaluated the catalytic performance of Au@PpTOC relative to a conventional homogeneous Au (III) catalyst (HAuCl_4_·3H_2_O) in the sequential alkylation–annulation reaction of 2,4‐dimethoxyaniline with propargyl bromide (see Supporting Information for experimental procedures; Table 2). The yields of the reactions were calculated based on isolated yields. It is important to note that the solvent plays a critical role in this reaction when using homogeneous catalysts. While the use of a nonpolar, aprotic solvent promotes the selective formation of an alkylation product, switching to ethanol, a polar protic solvent, enables the formation of the corresponding quinoline derivative. In agreement with the literature reports [18], homogeneous

**TABLE 2:** Comparison of the catalytic activity of homogeneous gold catalyst and Au@PpTOC in the alkylation and annulation reaction(s).

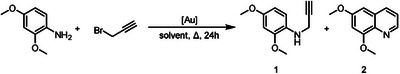				
Catalyst (10%)	Solvent	Temperature (°C)	1 Isolated Yield	2 Isolated Yield
PpTOC	Toluene	100	60	—
PpTOC	EtOH	80	43	52
HAuCl_4_.3H_2_O	Toluene	100	46	—
HAuCl_4_.3H_2_O	EtOH	80	—	60

*Note*: 1,4‐dimethoxyaniline and propargyl bromide was dissolved in 2 mL related solvent under argon and then the gold catalyst was added. The solution was heated to target temperature and stirred for 24 h, after which the product was isolated.

HAuCl_4_·3H_2_O catalyst led to the formation of alkylation product (**1**) with 46% yield in toluene at 100°C. On the other hand, we observed the formation of quinoline in 60% yield at 80°C using ethanol as the solvent. Proton transfer plays a crucial role in the catalytic cycle of the gold system. This transfer may be mediated by the solvent, counterions, or other nucleophiles present in the reaction [29, 30]. In EtOH, solvent‐assisted proton transfer promotes the annulation step leading to quinoline formation [31]. We selectively isolated the alkylation product **1** in the presence of Au@PpTOC when toluene was used as the solvent for the reaction in 60% yield, which was found to be superior compared to the homogeneous counterpart. Switching to EtOH enabled the change in the product selectivity, where Au@PpTOC showed the formation of corresponding quinoline **2** in 52% yield, which can be further increased by increasing the reaction time, along with an alkylation product (43%). This result shows the divergent product selectivity using the same heterogeneous catalyst by simply changing the solvent. Because gold is present in three oxidation states (Au(0), Au(I), and Au(III)) in Au@PpTOC after capture and partial reduction, its catalytic performance depends on the relative distribution of these species. Previous studies by Alfonsi et al. [19] showed that, under homogeneous conditions in ethanol, both Au(I) and Au(III) catalysts selectively produce the annulation product **2**, with higher yields observed for Au(III). In contrast, in the Au@PpTOC, a significant fraction of the gold is reduced to Au(0) within the polymer cavities, resulting in lower amounts of the catalytically active Au(I) and Au(III) species and introducing steric constraints that may slow the conversion of the alkylation intermediate **1** into the annulation product **2**. After the catalytic experiment, we investigated the changes in the distribution of Au species. Therefore, XPS measurements before and after catalysis were performed (Figures S23 and S24). The most notable changes can be observed in the Au4f spectra and deconvolutions (Figures S24D and H), where no suitable fit for Au(III) species could be made, while Au(0) and Au(I) become the predominant species. Nevertheless, extended reaction times lead to complete conversion of **1** to **2**, and the oxidation‐state distribution can, in principle, be tuned to further optimize catalytic performance. While solvent effects are well recognized in heterogeneous catalysis, they mostly influence reaction rates or adsorption equilibria rather than enabling access to distinct reaction pathways. In this context, Au@PpTOC showed a unique solvent‐controlled product divergence, with a single heterogeneous catalyst.

## 3 Conclusion

In summary, we report a phenazine‐derived tetraoxa[8]circulene‐based porous organic polymer that combines a crown ether cavity, high chemical stability, large surface area, and a heteroatom‐rich framework, enabling efficient and selective gold capture from aqueous media, including complex electronic waste solutions. The recovered polymer containing gold can be directly employed as a heterogeneous catalyst, thereby combining precious metal recovery with value‐added chemical transformations. Notably, we demonstrated solvent‐controlled product divergence using the same heterogeneous catalyst, switching between alkylation and annulation pathways without altering the catalyst composition. By combining selective gold recovery from electronic waste with tunable catalytic reactivity, this system demonstrates a circular approach in which waste‐derived precious metals are directly converted into functional heterogeneous catalysts for the synthesis of pharmaceutically relevant heterocycles. These results also showcase the potential of porous organic polymers as platforms for precious metal recovery and heterogeneous catalysis.

## Conflicts of Interest

The authors declare no conflicts of interest.

## Supporting information




**Supporting File 1**: The authors have cited additional references within the Supporting Information [32, 33, 34, 35, 36].

## Data Availability

The data that support the findings of this study are available on  Zenodo (10.5281/zenodo.19427184).
