# Supplementary material for: Porous Phenazine‐bridged Tetraoxa[8]Circulenes for Selective Gold Recovery and Heterogeneous Catalysis
Source: Angew Chem Int Ed Engl. 2026 Apr 8;65(22):e6586260. doi: 10.1002/anie.6586260 (PMC13206295; doi:10.1002/anie.6586260)
Supplement: Supplementary file 1 — Supporting File 1: The authors have cited additional references within the Supporting Information [32, 33, 34, 35, 36]. [file ANIE-65-e6586260-s001.docx]

**Characterization**

**Fourier-Transform infrared spectroscopy:** FTIR spectra were recorded on a PerkinElmer Frontier spectrometer equipped with a PIKE GladiATR module in the range of 4000-400 cm^–1^ at a scanning rate of 2 cm^–1^.

**Liquid-phase NMR spectra:** NMR spectra were recorded on a Bruker Ascend 400 MHz spectrometer using deuterated solvents. Values are given in ppm.

**Solid-state NMR spectra:** CP-MAS ^13^C solid-state NMR spectra were recorded on a Bruker Avance Neo 400 MHz spectrometer using 10 kHz spinning rate and a 5.0 s relaxation delay.

**Powder X-ray diffraction:** PXRD patterns were recorded on a Bruker D8 Advance system using Cu Kα1 and Kα2 incident beam in reflectance mode or, where noted, on a STOE Stadi-P in transmission mode. The diffractograms were recorded between 2θ 2-60° at 0.05°/step and a step time of 0.5s using a LYNXEYE XE-T detector. Transmission diffractograms were recorded in the range of 2θ 2 – 113.72 ° at 0.015 °/step.

**Thermogravimetric analysis:** TGA analysis was recorded on a Mettler-Toledo TGA/DSC 3+ instrument using standard 70 μL alumina crucibles. The flow rate of the gas (air or nitrogen) was set to 10 mL/min, and the heating rate was set to 10 °C/min for the 1000 °C. On average, the samples showed water uptake under ambient conditions of around 10%.

**Brunauer-Emmett-Teller surface area analysis:** Nitrogen sorption measurements were performed at 77K using a Micromeritics 3Flex instrument, after activation of the samples at 373K for 24h.The specific surface areas of the samples were calculated using the BET and Langmuir models in the pressure range where the term V(1- P/P_o_) continuously increases with P/P_o_ in the Rouquerol plot.

**X-ray photoelectron spectroscopy:** XPS was performed on a multi-purpose XPS (Sigma Probe, Thermo VG Scientific, X-ray Source: monochromatic Al Kα). ). All samples were prepared on an indium foil to reduce charging effects. However, indium signals are clearly visible for all measured samples in the XPS surveys, and indium oxides (denoted as InOx) are present in O1s deconvolutions.

**Inductively Coupled Plasma Optical Emission Spectroscopy**: ICP-OES analysis was performed using an OPTIMA7000 DV. Triplicates of each sample were measured and directly averaged on the machine. Between samples, the system was flushed with 5% nitric acid to remove impurities.

**Materials:**

1,4-dimethoxybenzene (99+%), aluminum chloride (anhydrous, 99.9%), and sodium chloride (99%) were purchased from Thermo Fisher Scientific. Glacial acetic acid (>99.7%) and sulfuric acid (>95%) were purchased from Fisher Scientific. Fuming nitric acid (100%) was purchased from Honeywell. Gold(III) chloride trihydrate, Palladium on carbon (5%), Celite 545, potassium tert-butyrate (98%), and boron tribromide (98%) were purchased from Sigma Aldrich. Sodium periodate was purchased from Fluorochem. Propargyl Bromide (80% in Toluene) and 2,4-Dimethoxyaniline >98.0% were purchased from TCI.

Solvents were, unless specified, used without any further purification. Ethyl acetate (EA), hexanes, methanol, and acetone were purchased from Thommen Furler. Toluene and dichloromethane were purchased from Thommen Furler and were dried in-house.**
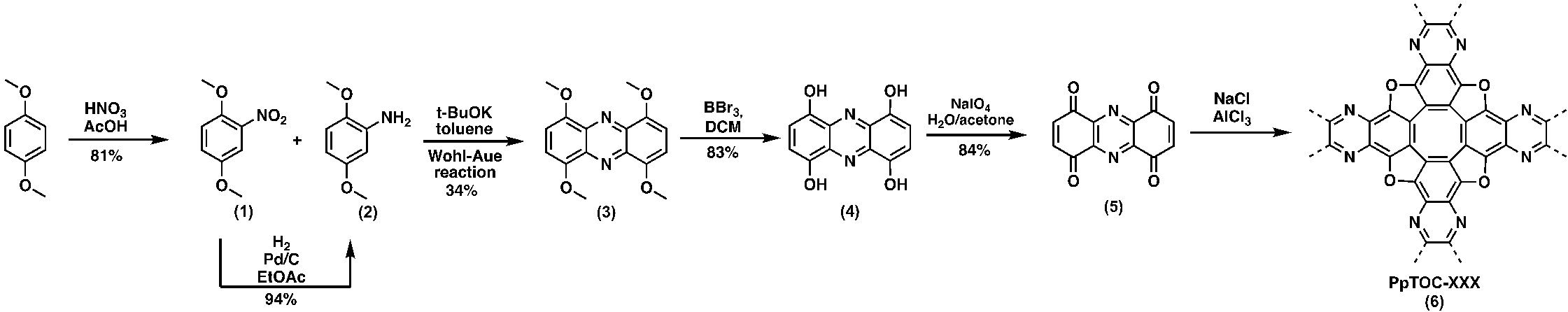
**

**Scheme S1**. Synthesis of polymeric phenazine-based tetraoxa[8]circulenes starting from 1,4-dimethoxybenzene.

**Synthesis of 2,5-dimethoxy nitrobenzene (1)**

A 250 mL two-neck flask was charged with 1,4-dimethoxybenzene (50.0 g, 361.8 mmol, 1.0 eq.) and 30 mL of glacial acetic acid before cooling to 0-10 °C with an ice bath. At this temperature, a mixture of fuming nitric acid (16.2 mL, 361.8 mmol, 1.0 eq.) in 30 mL glacial acetic acid was added dropwise over 1 hour. Subsequently, the yellow suspension was stirred for 1 hour at 0 °C, warmed to room temperature, and stirred for another 2 hours. After completion, the mixture was poured on ice, dissolved in 1.5 L of diethyl ether, and washed 3 times with ice-cold water. The organic phases were dried over MgSO_4_, and the solvent was removed, resulting in a yellow solid.

54.8 g (81%), yellow solid

^1^H NMR (400 MHz, chloroform-*d*) δ 7.38 (d, J = 3.1 Hz, 1H), 7.11 (ddd, J = 9.1, 3.0, 0.7 Hz, 1H), 7.03 (d, J = 9.2 Hz 1H), 3.91 (d, J = 0.7 Hz, 3H), 3.81 (d, J = 0.7 Hz, 3H)

Note: In case of impurities, the product was recrystallized from acetic acid.

**Synthesis of 2,5-dimethoxy aniline (2)**

An autoclave vial was charged with 20.0 g of 2,5-dimethoxy nitrobenzene, 0.50 g 10% Pd/C, and 50 mL of ethyl acetate. The vial was backfilled three times with hydrogen and finally pressurized with 40 bars of hydrogen gas, stirred at room temperature, and refilled with hydrogen if necessary. The completion of the reaction was checked with TLC. After completion, the contents of the vial were filtered over Celite, and after washing with additional ethyl acetate, the solvent was removed.

15.7 g (94%), grey-white solid

^1^H NMR (400 MHz, chloroform-*d*) δ 6.70 (d, J = 8.7 Hz, 1H), 6.33 (d, J = 2.9 Hz, 1H), 6.24 (dd, J = 8.7, 2.9 Hz 1H), 3.81 (d, J = 1.1 Hz, 3H), 3.73 (s, 3H) ppm.

**Synthesis of 1,4,5,8-tetramethoxy phenazine (3)**

Procedure adapted from Conboy et al. ^[32]^

A 1000 mL two-neck flask was flushed with argon and charged with 2,5-dimethoxy nitrobenzene (35.0 g, 191.1 mmol, 3.0 eq.), 2,5-dimethoxy aniline (9.75 g, 63.7 mmol, 1.0 eq.), potassium *tert*-butoxide (28.5 g, 254.8 mmol, 4.0 eq.), and 600 mL of dry toluene. The reaction mixture was heated to 110 °C for 48 hours under vigorous stirring. After cooling to room temperature, the contents were filtered over a large POR 4 fritted glass filter and washed extensively with water. The obtained solid was dried, ground with a mortar, and triturated from DCM.

6.5 g (34%), red-orange solid

^1^H NMR (400 MHz, chloroform-*d*) δ 6.97 (s, 4H), 4.08 (s, 12H) ppm.

^13^C NMR (100 MHz, chloroform-*d*) δ 149.01, 135.80, 106.10, 56.18 ppm/

**Note**: The trituration step may be repeated several times to improve the purity of the product.

**Synthesis of 1,4,5,8-tetra hydroxy phenazine (4)**

A 1000 mL two-neck flask was flushed with argon and charged with 1,4,5,8-tetra methoxy phenazine (6.5 g, 21.6 mmol, 1.0 eq.) and 500 mL of dry DCM. The flask was cooled to 0 °C before adding BBr_3_ (16.3 mL, 43.3 g, 173.2 mmol, 8.0 eq., 2 eq. per OMe group) over the course of 1 hour. The mixture was stirred at 0 °C for 1 hour before warming to room temperature and stirring overnight. The reaction was cooled to 0 °C and slowly quenched with water, resulting in a purple suspension. The suspension was filtered over a POR 4 fritted glass filter and washed extensively with water. The product was dried and obtained as a dark purple powder.

4.3 g (83%), purple solid

^1^H NMR (400 MHz, DMSO-*d_6_*) δ 9.53 (s, 4H), 7.08 (s, 4H) ppm.

^13^C NMR (100 MHz, DMSO-*d_6_*) δ 145.48, 133.24, 110.45 ppm.

**Note**: In case the product was not obtained pure, the powder was ground with a mortar and stirred in refluxing DCM overnight to remove unreacted byproducts. After filtering over a POR 4 fritted glass filter, the powder was washed with DCM and dried under vacuum.

**Synthesis of 1,4,5,8-phenazine tetraone (5)**

A 100 mL two-neck flask was charged with 1,4,5,8-tetrahydroxy phenazine (3.5 g, 14.3 mmol, 1.0 eq.). The solid was suspended in 50 mL of water and 10 mL of acetone before adding sodium periodate (13,4 g, 63.0 mmol, 4.4 eq.) and one drop of concentrated sulfuric acid. The mixture was stirred vigorously overnight under air. The obtained brown suspension was poured on 150 mL of distilled water and filtered over a POR 4 fritted glass filter, washed with water, and dried under vacuum.

2.9 g (84%), brown solid

^1^H NMR (400 MHz, DMSO-*d_6_*) δ 7.39 (s, 4H) ppm.

^13^C NMR (100 MHz, DMSO-*d_6_*) δ 182.37, 145.77, 139.91 ppm.

**Note**: In case of impurities, the product was triturated from DCM.

**Note**: Direct oxidation of (3) with ceric ammonium nitrate supported on silica (5.5 eq.) was attempted but did not result in the desired product. Oxidation of (5) with PIFA (4.4 eq.) was also attempted, but did not result in the desired product.

**Synthesis of phenazine-based tetraoxa[8]circulenes (6)**

In a glovebox, 1,4,5,8-phenazine tetraone (600 mg, 2.50 mmol, 1.00 eq.), aluminum chloride (933 mg, 7.00 mmol, 2.80 eq.), and sodium chloride (257 mg, 4.40 mmol, 1.76 eq.) were ground together in a mortar. The ground powder was transferred to a thick-walled ampoule (85 x 20 x 1 mm, height x width x wall thickness). The ampoule was capped with parafilm and transferred outside the glovebox and evacuated for 45 minutes to 0.01 mbar. The ampoule was sealed and heated to 250/300/350 °C (for PpTOC-250, -300, -350 respectively; 100 °C per hour heating rate) for 72 hours. After cooling to room temperature, the ampoules were opened, and the contents were quenched with water. The dark brown powder was filtered over a POR 4 fritted glass filter and washed with water, pyridine, and methanol. Subsequently, the powder was stirred in 600 mL of water overnight, filtered again, and washed with pyridine, methanol, and hexanes. The powder was suspended in hexane, which was slowly evaporated at ambient conditions. The product was further dried at <0.01 mbar at 90 °C, and the product was obtained as a dark brown powder.

0.501 g (98%), dark brown powder

**Note**: It is advised to use thick-walled ampoules as internal pressures can build up and result in vial ruptures.

**Note**: During all washing steps, special care was taken to avoid drying the powder under any circumstances. Our experiments have shown that conventional drying results in vastly reduced BET surface areas.

**Au uptake experiments:**

Two types of stock solutions were used for the experiments.

Method A: 1000 ppm Au stock solutions purchased from Sigma Aldrich were used directly or diluted to the desired concentration using 2% nitric acid.

Method B: Stock solutions of the desired concentration were prepared by weighing HAuCl_4_*3H_2_O and dissolving the salt with double-distilled water.

In both cases, the exact concentration of the solutions was determined on each day on which experiments were performed.

All experiments were performed at least in triplicate.

**Maximum uptake**

A 25 mL Schott flask was charged with 5 or 10 mg of PpTOC-350 before adding 10 mL of stock solution (Method A and B). The flask was closed, and the solution stirred at 350 rpm for 24 hours, after which the solution was filtered through a 0.45 µm PTFE filter and analyzed using ICP-OES.

| $Gold adsorption \left( mg_{Au} g_{Polymer}^{-1} \right)=\frac{Q_{i}-Q_{e}}{m_{Polymer}}$ | (1) |
| --- | --- |

Where *Q*_i_ is the amount of gold in the initial solution, *Q*_e_ is the amount of gold after the uptake experiment, and *m*_Polymer_ is the mass of the polymer used in the experiment.

The uptake isotherm was fitted according to the Langmuir adsorption model, following equation (2):

| $Q_{e}=\frac{Q_{m}*K_{L}*C_{e}}{1+K_{L}*C_{e}}$ | (2) |
| --- | --- |

where *Q*_e_ (*g*_Au_/*g*_Polymer_) is the quantity of adsorbed metal ions in a gram of adsorbent at equilibrium, *C*_e_ (mg L^–1^) is the equilibrium concentration, *Q*_m_ (*g*_Au_/*g*_Polymer_) is the maximum uptake amounts of metal ions in a gram of adsorbent, and *K*_L_ is the Langmuir constant.

**Uptake kinetics**

A 100 mL Schott flask was charged with 100 mg of PpTOC-350 and 100 mL of a 500 ppb or 25 ppm Au stock solution (Method A). The solution was stirred at 350 rpm, and aliquots were taken after specified periods of time. The aliquots were filtered through a 0.45 µm PTFE filter and analyzed using ICP-OES.

Pseudo-first-order kinetics:

| $Q_{t}=Q_{e}\left( 1-e^{k_{1}*t} \right)$ | (3) |
| --- | --- |

Where *Q*_t_ is the amount of Au adsorbed at time *t*, *Q*_e_ is the amount of Au adsorbed at equilibrium, and *k*_1_ is the pseudo-first-order rate constant (min^-1^).

Pseudo-second-order kinetics:

| $Q_{t}=\frac{Q_{e}^{2}*k_{2}*t}{1+{Q_{e}*k}_{2}*t}$ | (3) |
| --- | --- |

Where *Q*_t_ is the amount of Au adsorbed at time *t*, *Q*_e_ is the amount of Au adsorbed at equilibrium, and *k*_2_ is the pseudo-second-order rate constant (g mg^-1^ min^-1^).

**pH adjustment experiments**

Before each pH experiment, the pH meter was calibrated using commercially available pH 4 and pH 7 standards.

A 1000 ppm stock solution was prepared from HAuCl_4_*3H_2_O using double-distilled water to dissolve the salt (Method B). The pH of the solution was determined to be 2.5. Under stirring, the pH of the solution was adjusted using small amounts of 25% aqueous ammonia or 2N NaOH. At specific pH values, aliquots were taken and added to prepared 25 mL Schott flasks containing PpTOC-350. The solutions were stirred at 350 rpm for 24 hours, after which the solutions were filtered using 0.45 µm PTFE filters and analyzed using ICP-OES.

Additionally, the stability of these Au solutions was determined by separating an aliquot and adding it to a separate vial.

**Polymer recycling experiments**

A 100 mL Schott flask was charged with 50 mg of PpTOC-350 and 50 mL of a 25 ppm Au stock solution (Method A). The solution was stirred at 350 rpm for 3 hours, after which the solution was filtered using a POR4 fritted glass filter. Aliquots of the obtained solution were used to determine the residual Au concentration using ICP-OES. The polymer was briefly dried and then transferred to a 100 mL Schott flask and treated with 70 mL of a 1:1 mixture of 1M thiourea and 1M HCl for 18 hours. The polymer was filtered using another POR4 fritted glass filter, washed with water, and used for subsequent uptake experiments.

**Au uptake under competitive conditions**

Using 1000 ppm stock solutions, a mixed metal solution (40 ppm and 50 ppm) containing Au, Re, Co, Cu, Pd, and Rh was prepared. This solution was added to 25 mL Schott flasks containing PpTOC-350. The solution was stirred at 350 rpm for 24 hours, after which the solutions were filtered using 0.45 µm PTFE filters and analyzed using ICP-OES.

**Au recycling from spent electronics**

Printed circuit boards (random access memory kits and graphics cards) were sourced from a landfill. PCBs were suspended in a solution of NBS/pyridine (1.42 g NBS and 0.64 mL pyridine in 750 mL double-distilled water) and soaked for 72 hours. After removing larger parts, the solution was filtered and pH was adjusted to 2.5 by using 1M HCl solution.

For the uptake experiments, 25 mL Schott flasks were charged with 10 mg of PpTOC-350 and 10 mL of the leach solution. After stirring for 4 h at 350 rpm, the solutions were filtered using a 0.45 µm PTFE filter and analyzed using ICP-OES.

**Table S1**. Metal ions of interest were found in the solution obtained after the computer electronics leaching experiment.

| Metal | Concentration (mg L^-1^) |
| --- | --- |
| Ni | 0.724 |
| Si | 0.08 |
| Au | 4.579 |
| Cu | 368.5 |
| Pd | 0.027 |

**Activated carbon experiments**

A 25 mL Schott flask was charged with 10 mg of activated carbon before adding 10 mL of stock solution (Method A). The flask was closed, and the solution was stirred at 350 rpm for 24 hours, after which the solution was filtered through a 0.45 µm PTFE filter and analyzed using ICP-OES.

Additionally, mixed-metal uptake experiments were performed in the same manner as for PpTOC.

**Gold uptake under irradiation**

A stock solution containing 1,270 ppm of gold was prepared by weighing out HAuCl₄·3H₂O and dissolving the salt in double-distilled water. The pH of the stock solution was adjusted to 2.5 by 1M HCl solution. Then, two flasks were charged with 50 mg of PpTOC and 50 mL of the stock solution, respectively. One flask was placed under a halogen lamp, while the other was covered with an aluminum foil and placed in dark conditions. The flasks were closed, and the solutions were stirred at 350 rpm for 72 hours. Then, the solutions were filtered through a 0.45 µm PTFE filter and analyzed using ICP-OES.

**Homogeneous and Heterogeneous Gold Catalysis Experiments^[18]^**


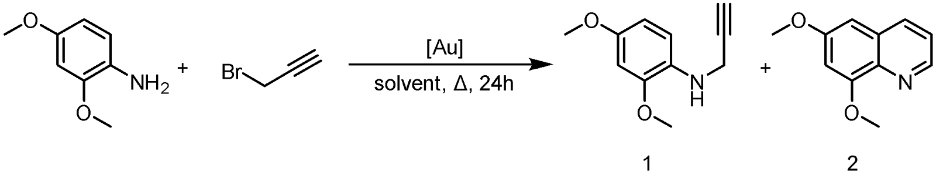


Procedure **(P1):** A 25 mL round-bottom flask was charged with 153 mg (1 mmol, 3 equivalents) of 2,4-dimethoxyaniline under argon and dissolved in 2 mL of toluene. Then, 28.5 μL (333 μmol, 1 equiv) of propargyl bromide and the appropriate 10% gold catalyst were added. The reaction mixture was stirred at 100°C for 24 hours. After cooling to room temperature, the toluene was removed under reduced pressure, and the slurry was dissolved in 100 mL of EtOAc. The organic solution was extracted three times with 100 mL of a saturated NaHCO₃ solution, and then the aqueous phase was washed with 100 mL of EtOAc. The combined organic phase was dried over MgSO₄ and concentrated under reduced pressure. The residue was purified by flash chromatography (silica gel, n-hexane–EtOAc, 4:1) to produce the product, **P1.**

^1^H NMR (400 MHz, CDCl_3_): δ 6.64 (d, *J* = 8.4 Hz, 1H), 6.53 – 6.38 (m, 2H), 3.93 (d, *J* = 2.4 Hz, 2H), 3.83 (s, 3H), 3.77 (s, 3H), 2.20 (t, *J* = 2.4 Hz, 1H) ppm.

^13^C NMR (100 MHz, CDCl_3_) δ 152.96, 148.67, 131.11, 111.54, 103.79, 99.40, 81.63, 71.08, 55.90, 55.53, 34.12 ppm.

Procedure **(P2):** A 25 mL round-bottom flask was charged with 153 mg (1 mmol, 3 equivalents) of 2,4-dimethoxyaniline under argon and dissolved in 2 mL of ethanol. Then, 28.5 μL (333 μmol, 1 equiv) of propargyl bromide and the appropriate 10% gold catalyst were added. The reaction mixture was stirred at 100°C for 24 hours. After cooling to room temperature, ethanol was removed under reduced pressure, and the slurry was dissolved in 100 mL of EtOAc. The organic solution was extracted three times with 100 mL of a saturated NaHCO₃ solution, and then the aqueous phase was washed with 100 mL of EtOAc. The combined organic phase was dried over MgSO₄ and concentrated under reduced pressure. The residue was purified by flash chromatography (silica gel, DCM and then DCM with 5% MeOH) to produce the product, **P2.**

^1^H NMR (400 MHz, CDCl_3_) δ 8.76 (dd, *J* = 4.3, 1.7 Hz, 1H), 8.00 (dd, *J* = 8.4, 1.7 Hz, 1H), 7.37 (dd, *J* = 8.3, 4.3 Hz, 1H), 6.72 (d, *J* = 2.6 Hz, 1H), 6.65 (d, *J* = 2.4 Hz, 1H), 4.05 (s, 3H), 3.92 (s, 3H) ppm.

^13^C NMR (100 MHz, CDCl_3_) δ 158.47, 156.47, 146.85, 138.99, 134.97, 130.17, 122.29, 101.40, 97.00, 56.18, 55.66 ppm.


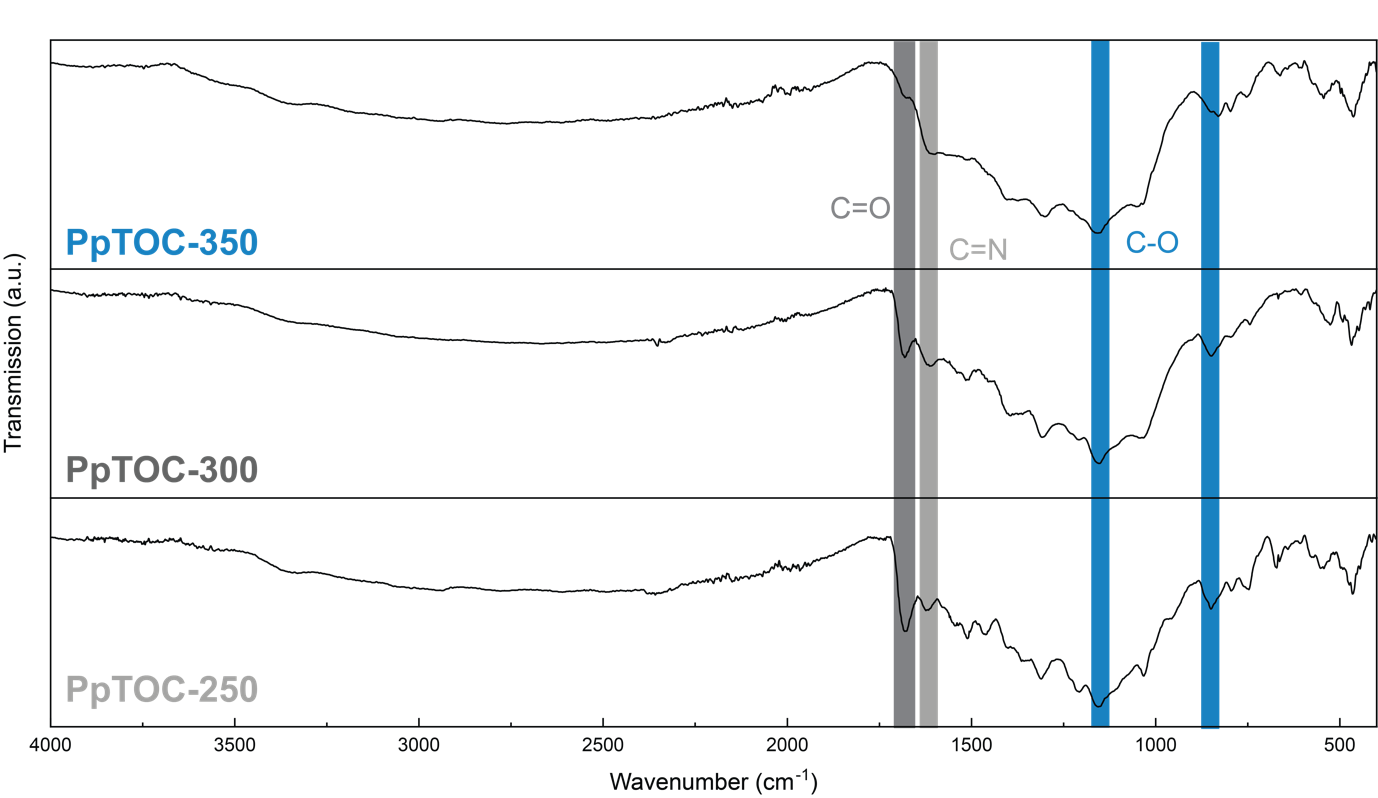


**Figure S1**. Fourier-transform infrared spectra of PpTOCs prepared at different temperatures. C=O modes are highlighted in dark grey, C=N modes in light grey, and C-O modes in blue.

**
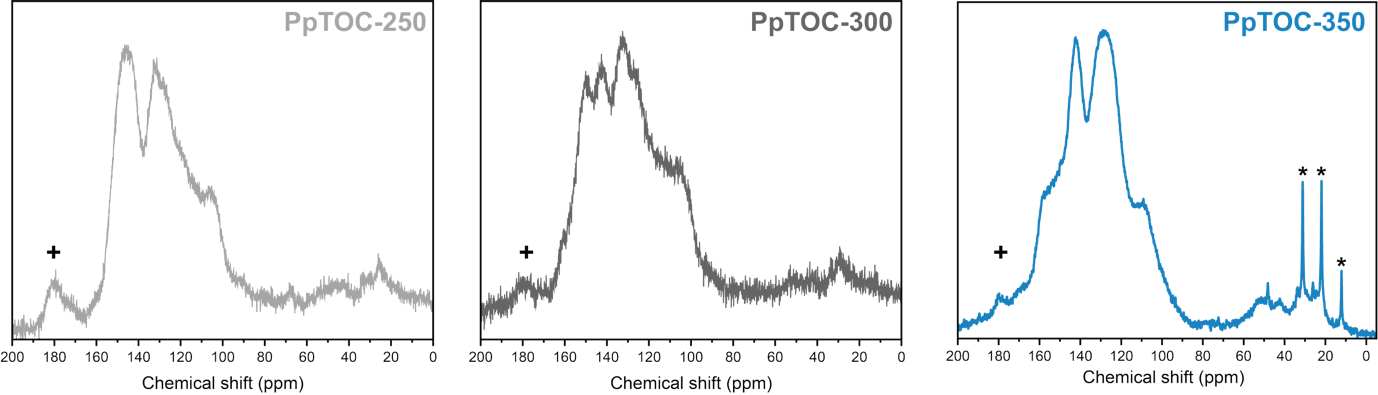
**

**Figure S2**. Cross-polarization magic angle spinning ^13^C NMR spectra of PpTOCs prepared at different reaction temperatures. Peaks highlighted with (+) correspond to residual unreacted/terminal carbonyls, while peaks highlighted with (*) are attributed to grease on the NMR rotor. Spectra were recorded at 10 kHz rotating speed and 100 MHz of field strength.


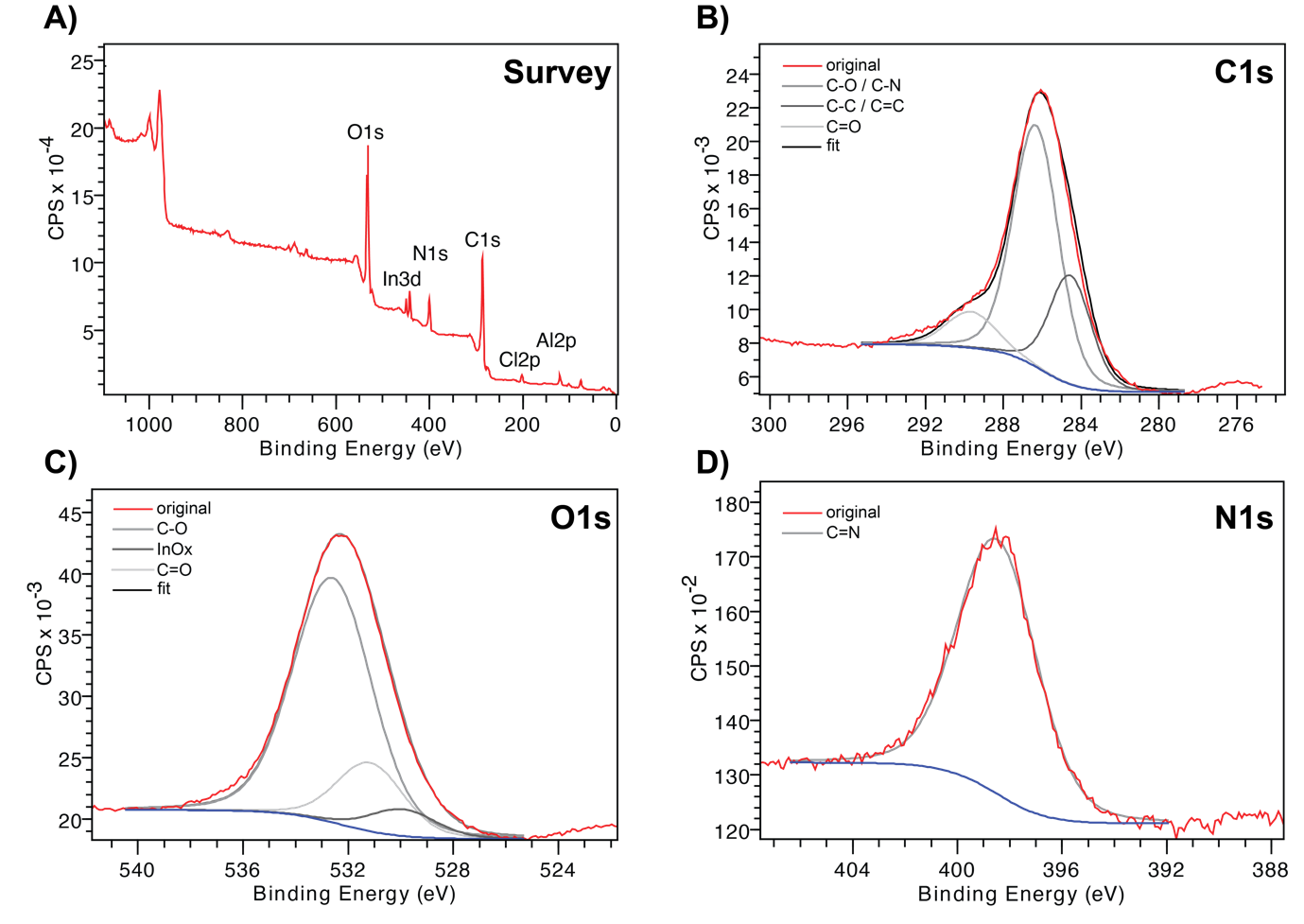


**Figure S3**. X-ray photoelectron spectra of PpTOC-350. A) Survey spectrum indicating the presence of carbon, nitrogen, oxygen, aluminum, and chlorine from the sample and indium from the support. B) C1s deconvolution, C) O1s deconvolution, and D) N1s deconvolution. Note: Due to the high abundance of C-O and C=N, all spectra were shifted to 286.0 eV instead of to the adventitious carbon peak at 284.8 eV.


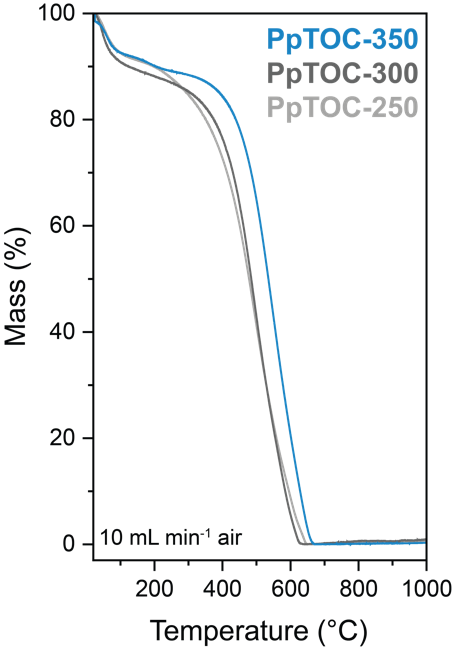


**Figure S4**. Thermogravimetric analysis of PpTOC prepared under different conditions was measured under an airflow. A notable water uptake under ambient conditions is visible by the mass loss at the beginning of the measurement. If samples were dried on the machine at 130 °C under a nitrogen flow, no early mass loss was observed.


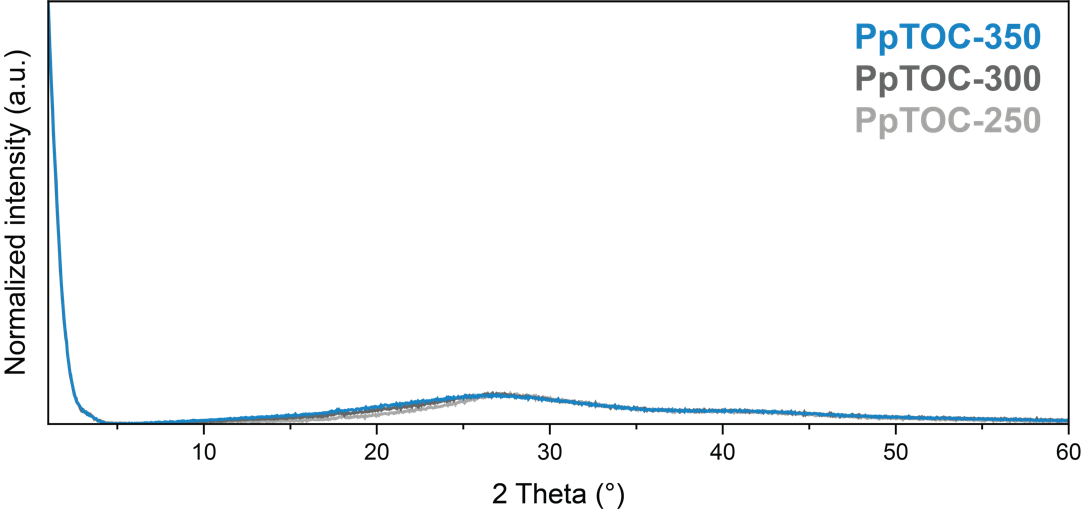


**Figure S5**. Powder X-ray diffractograms of PpTOCs prepared under different conditions. In all cases, amorphous polymers were obtained. The absence of sharp peaks originating from residual (metal)salts indicates the complete removal/low concentration, or amorphous character thereof.


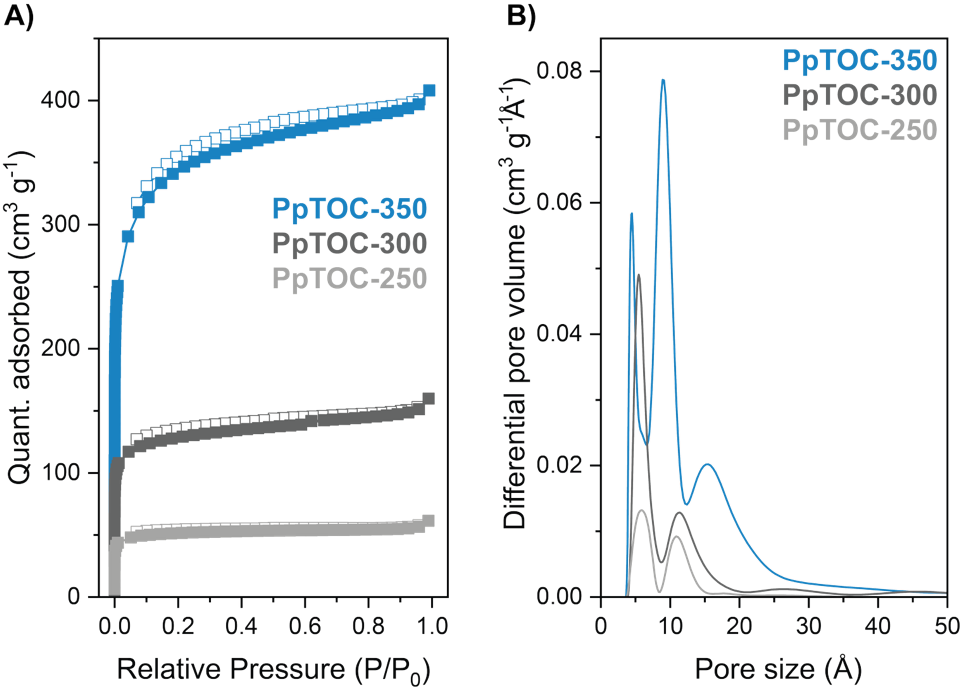


**Figure S6**. A) Brunauer-Emmett-Teller surface area plots for PpTOCs prepared at different temperatures and B) their NLDFT pore size analysis. Note: Two different NLDFT kernels were used for the determination of the pore size to minimize fitting errors. PpTOC-250 and PpTOC-300 were fitted using the carbon slit model and PpTOC-350 was fitted using the heterogeneous surface model.


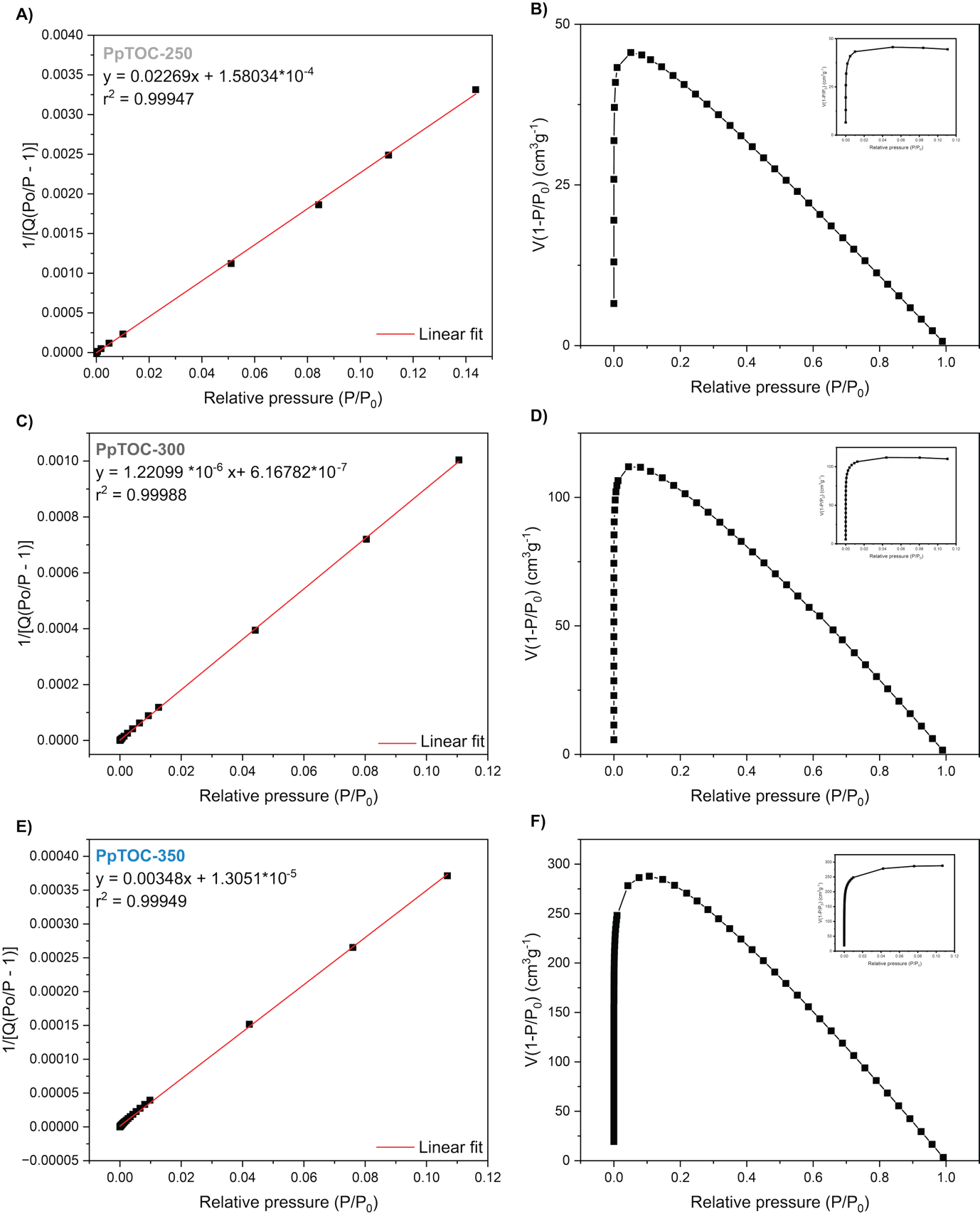


**Figure S7**. BET linear (A, C, E) and Rouquerol (B, D, F) plots of PpTOCs prepared at different temperatures. According to these plots, the valid pressure range for the calculation of the BET surface area was determined.


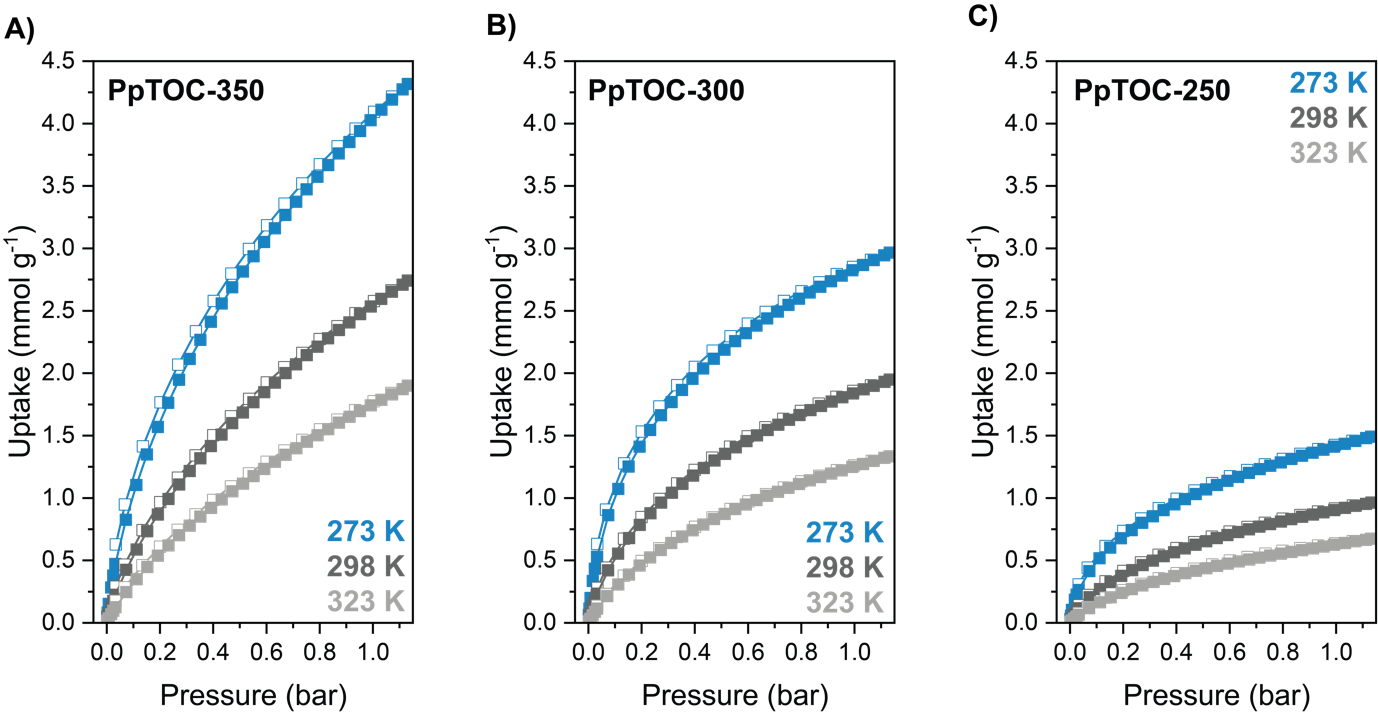


**Figure S8**. CO_2_ sorption performance of A) PpTOC-350, B) PpTOC-300, and C) PpTOC-250 performed at different temperatures. The substantial changes between the samples can be explained by their differences in BET surface area and, particularly, their accessible micropore volume.


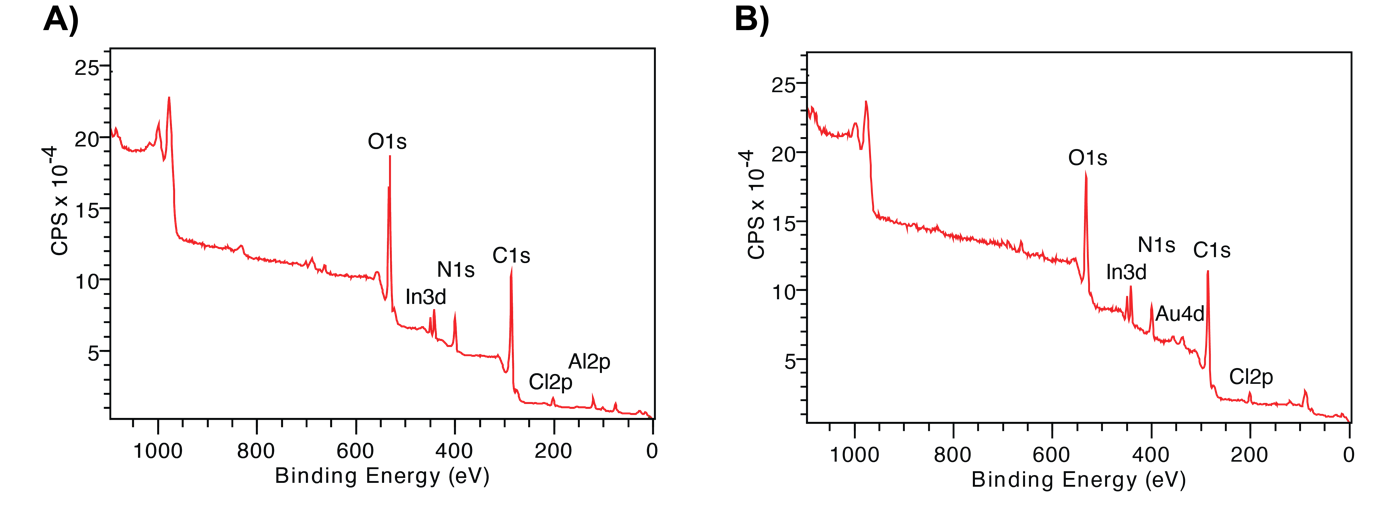


**Figure S9**. X-ray photoelectron spectra of A) PpTOC-350 and B) PpTOC-350 after gold uptake. Notably, no aluminum signals were detected, while Au signals are visible. Note: The samples were supported on an indium foil for the measurement. Thus, signals related to indium and its oxides (denoted as InOx) are present in survey spectra.


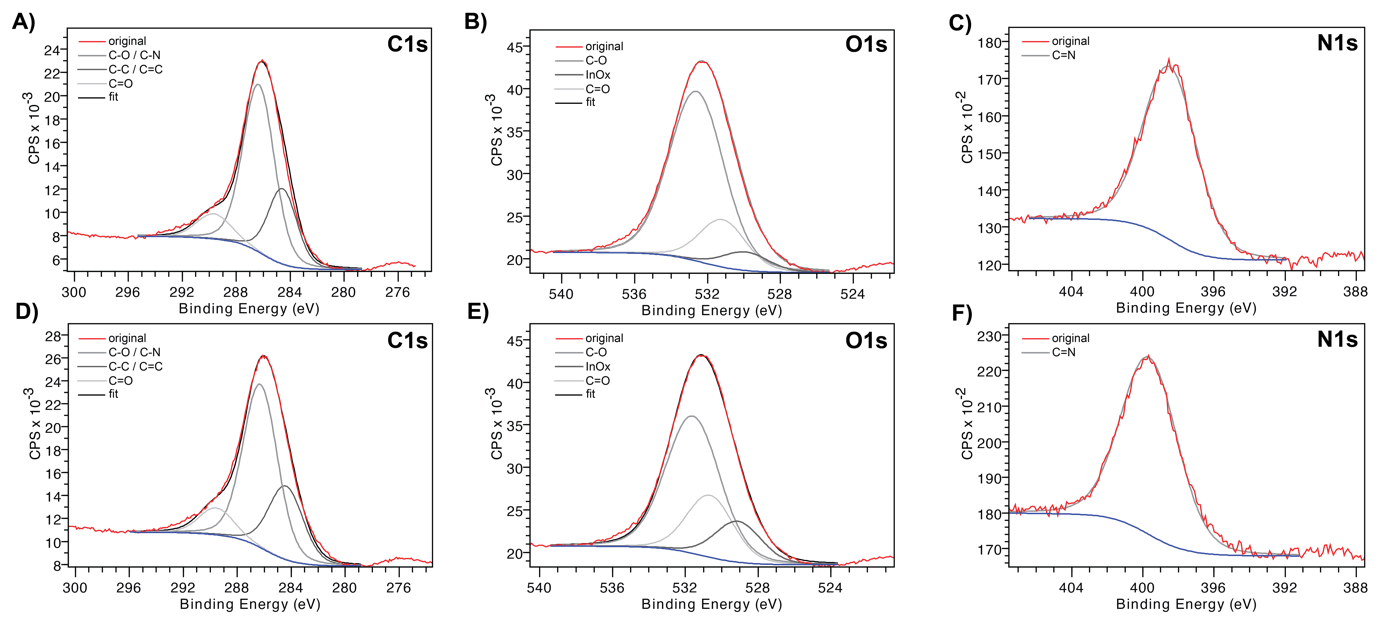


**Figure S10**. Deconvolutions of XPS narrow scans of (A – C) PpTOC-350 and (D - F) PpTOC-350 after gold uptake. While little change is observed in the C1s and O1s spectra, the N1s was shifted slightly to higher energies. Note: Due to the high abundance of C-O and C=N, all spectra were shifted to 286.0 eV instead of to the adventitious carbon peak at 284.8 eV. *Note 2: The samples were supported on an indium foil for the measurement. Thus, signals related to indium and its oxides (denoted as InOx) are present in the O1s deconvolutions.*


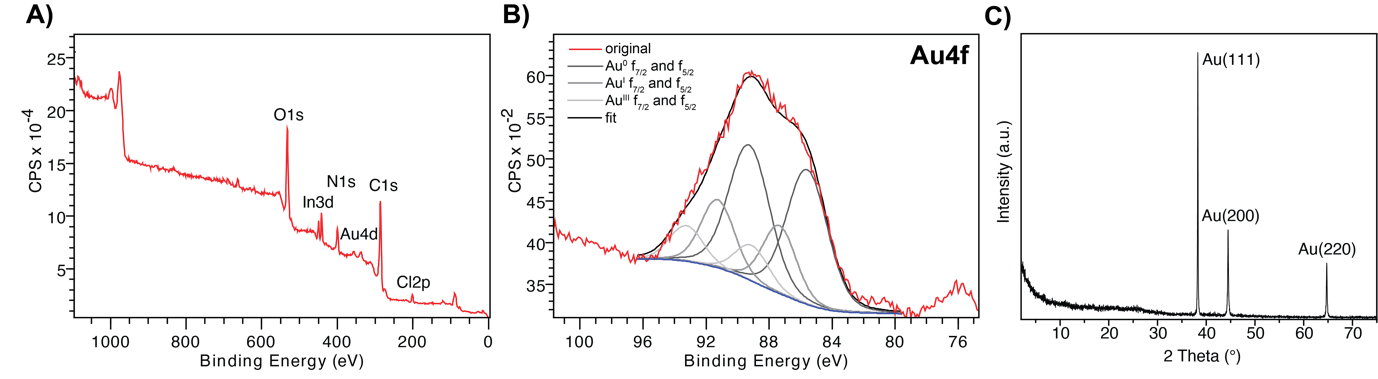


**Figure S11**. Analysis of PpTOC-350 after gold uptake. A) XPS survey showing the presence of gold and removal of Al, indicating that residual Cl likely originates from coordinated or partially reduced gold species. B) Deconvolution of the Au4f region. The broad peak indicates the presence of several gold species (Au(0), Au(I), and Au(III)). Note: Due to the high abundance of C-O and C=N, all spectra were shifted to 286.0 eV instead of to the adventitious carbon peak at 284.8 eV. C) Powder X-ray diffractogram showing the presence of elemental Au.


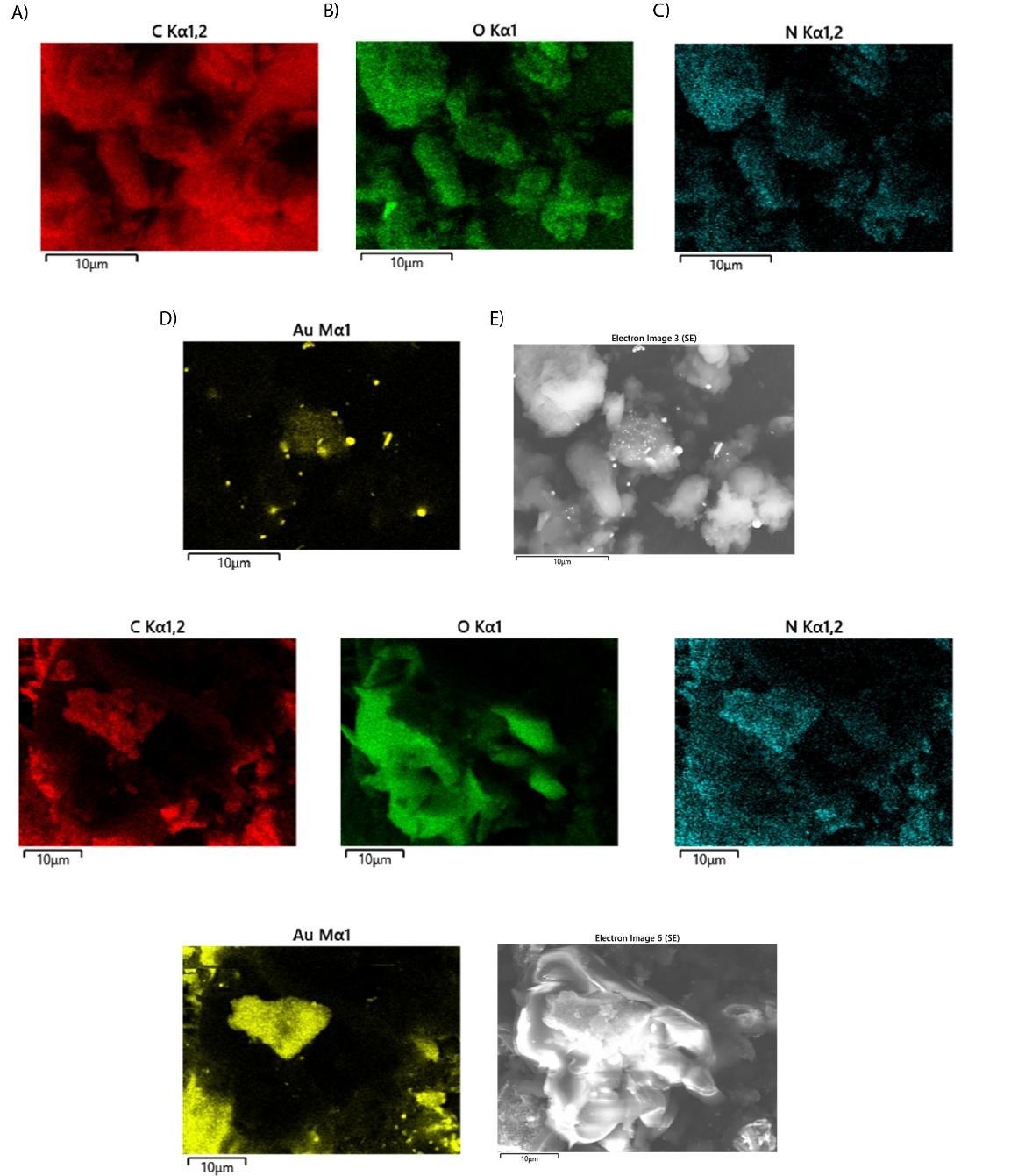


**Figure S12**. SEM images and EDX elemental mapping of PpTOC after gold uptake.


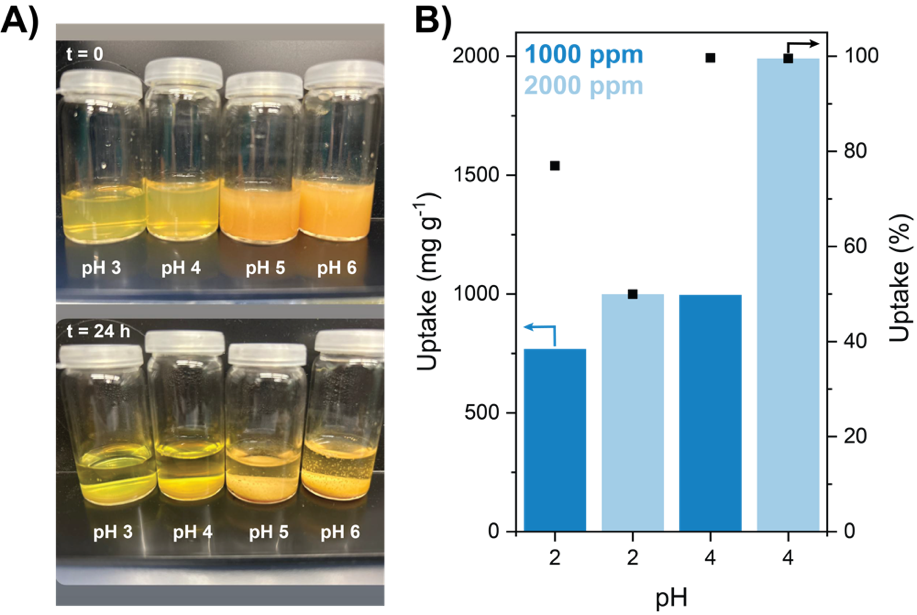


**Figure S13**. Effect of pH-adjustment on Au stock solutions and on the Au-uptake performance. A) 1000 ppm Au stock solution prepared from HAuCl_4_*3H_2_O and adjustment to different pH values using 25% ammonia solution after preparation (top) and after 24 h (bottom). The formation of precipitates is clearly visible for all pH-adjusted solutions right after preparation and after 24 hours. B) Results of uptake experiments performed at pH 2 and pH 4 with different stock solutions. While the experiment performed at pH 2 showed no precipitate formation and normal uptake performance, the experiment at pH 4 showed full uptake for both solutions. This was likely caused by partial or full precipitation of Au species.


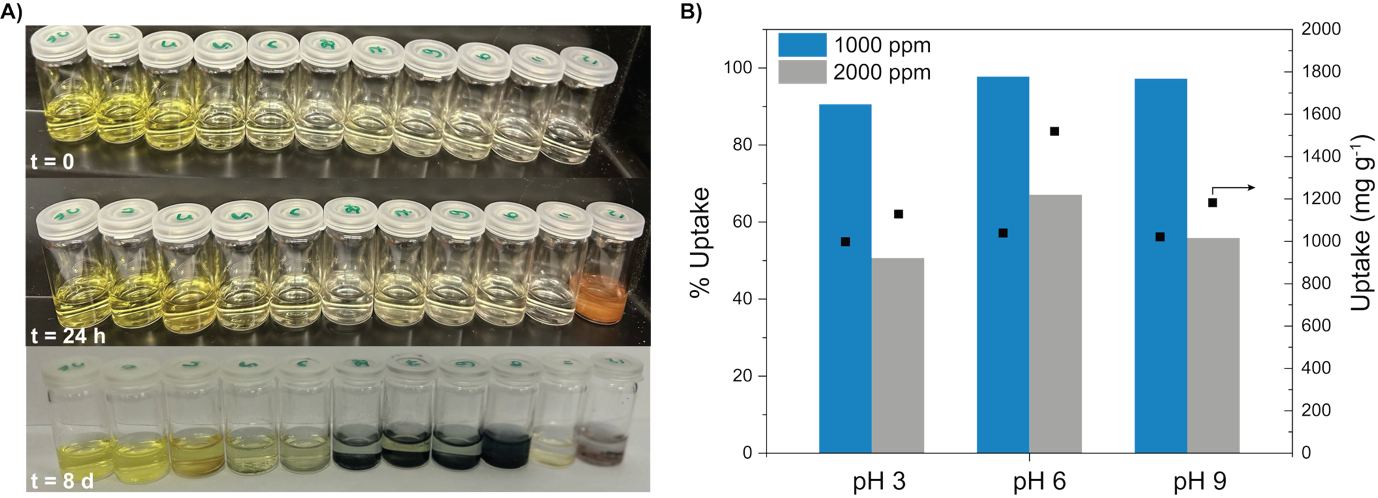


**Figure S14**. Effect of pH-adjustment on Au stock solutions with NaOH and on the Au-uptake performance. A) 1000 ppm Au stock solution prepared from HAuCl_4_*3H_2_O adjusted to different pH values using 2N NaOH after preparation (top), after 24 h (middle), and after 8 days (bottom) B) Results of uptake experiments performed at pH 3, pH 6, and pH 9 with different stock solutions. % Uptake shows the Au uptake based on the used stock solution.


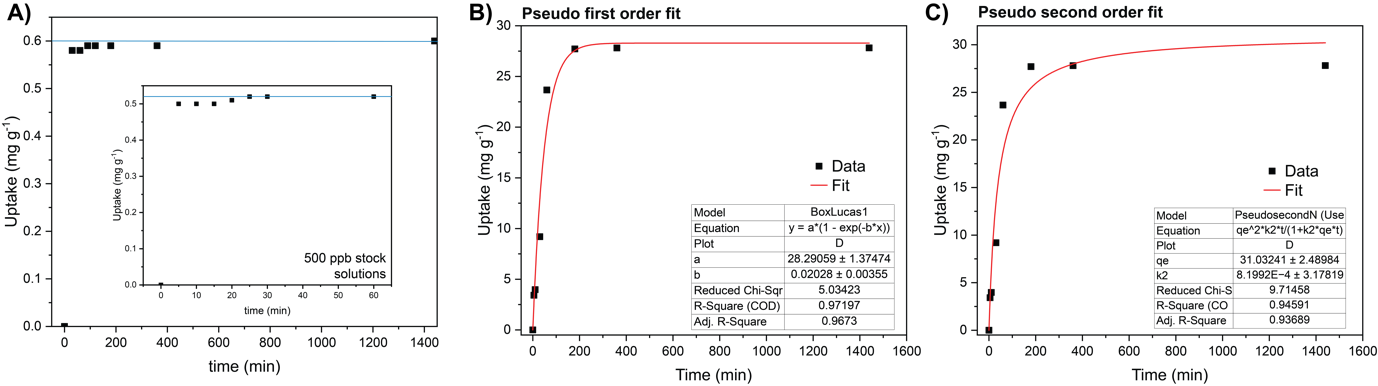


**Figure S15**. A) Gold uptake kinetics using a 500-ppb stock solution. Inset: Repeat experiment with aliquots taken at shorter intervals. Gold uptake kinetics using a 25-ppm stock solution with a B) pseudo-first-order and C) a pseudo-second-order fit.

**Table S2**. Comparison of uptake performance of gold adsorbents based on COFs and POPs along with the details of pH values at which the uptake experiments were performed.

| Material | Functionality | Uptake (g g^-1^) | pH | Reference |
| --- | --- | --- | --- | --- |
| COP-210 | Porphyrin | 1.176 | 2 | [33] |
| COP-211 | Porphyrin | 0.901 | 2 | [33] |
| COP-212 | Porphyrin | 1.250 | 2 | [33] |
| COP-180 | Porphyrin/Phenazine | 1.620 | 2 | [15] |
| COP-223 | Porphyrin/Phenazine | 1.087 | 2 | [16] |
| COP-224 | Porphyrin/Phenazine | 1.315 | 2 | [16] |
| BIT-POP-15 | N-phenylnorborene-polymer | 2.321 | 4 | [13] |
| BIT-POP-15 (light) | N-phenylnorborene-polymer | 3.020 | 4 | [13] |
| Ionic-COF-Cl | Ionic guanidinium COF | 1.271 | 2.5-4.5 | [12] |
| Ionic-COF-Br | Ionic guanidinium COF | 0.794 | 2.5-4.5 | [12] |
| Ionic-COF-SO4 | Ionic guanidinium COF | 0.758 | 2.5-4.5 | [12] |
| Ionic-COF-AcO | Ionic guanidinium COF | 0.694 | 2.5-4.5 | [12] |
| im-PYTA-PZDH-COF | imidazopyridinium COF | 1.558 | n.s. | [11] |
| im-PYTA-BPDH-COF | imidazopyridinium COF | 1.220 | n.s. | [11] |
| WDTA-TAB | amide POP | 2.040 | n.s. | [34] |
| TTB-COF | Thioether functionalized COF | 0.560 | n.s. | [35] |
| TTF-COF | TTF-COF | 1.639 | 5 | [17] |
| T-PAC | Thiophene-bearing cage | 2.260 | n.s. | [36] |
| **PpTOC-350^[a]^** | **Phenazine/ aza-crown ether** | **0.860** | **2.5** | **this work** |
| **PpTOC-350^[b]^** | **Phenazine/ aza-crown ether** | **1.256** | **2.5** | **this work** |
| **PpTOC-350** | **Phenazine/ aza-crown ether** | **0.948** | **2.5** | **this work** |
| ^[a] Under halogen lamp for 72 hours, [b] Dark conditions for 72 hours.^  ^n.s.: not specified^ | | | | |


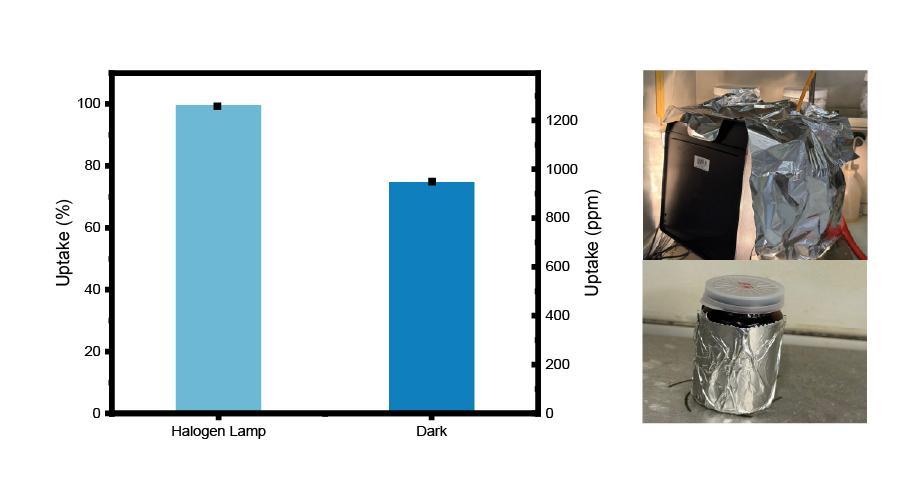


**Figure S16**. Comparison of gold uptake performance when samples were irradiated with a halogen lamp in comparison to when samples were kept in the dark. A) Au uptake performance of samples exposed to halogen lamp versus dark conditions. B) Experimental setups: halogen lamp (top) and dark environment (bottom).


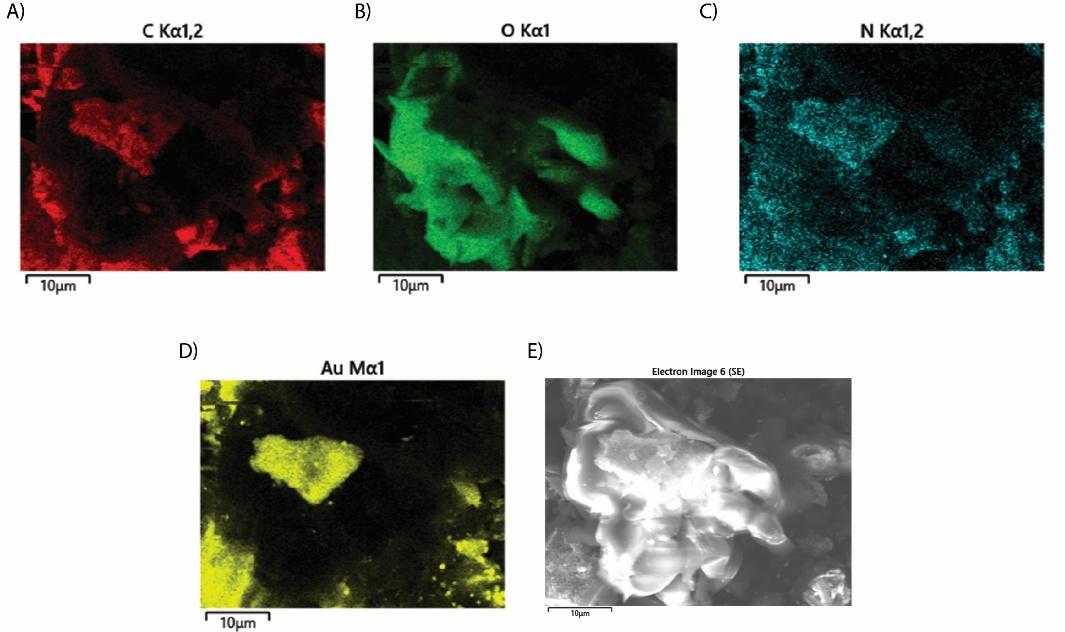


**Figure S17**. SEM images and EDX elemental mapping of PpTOC after gold uptake under halogen lamp.


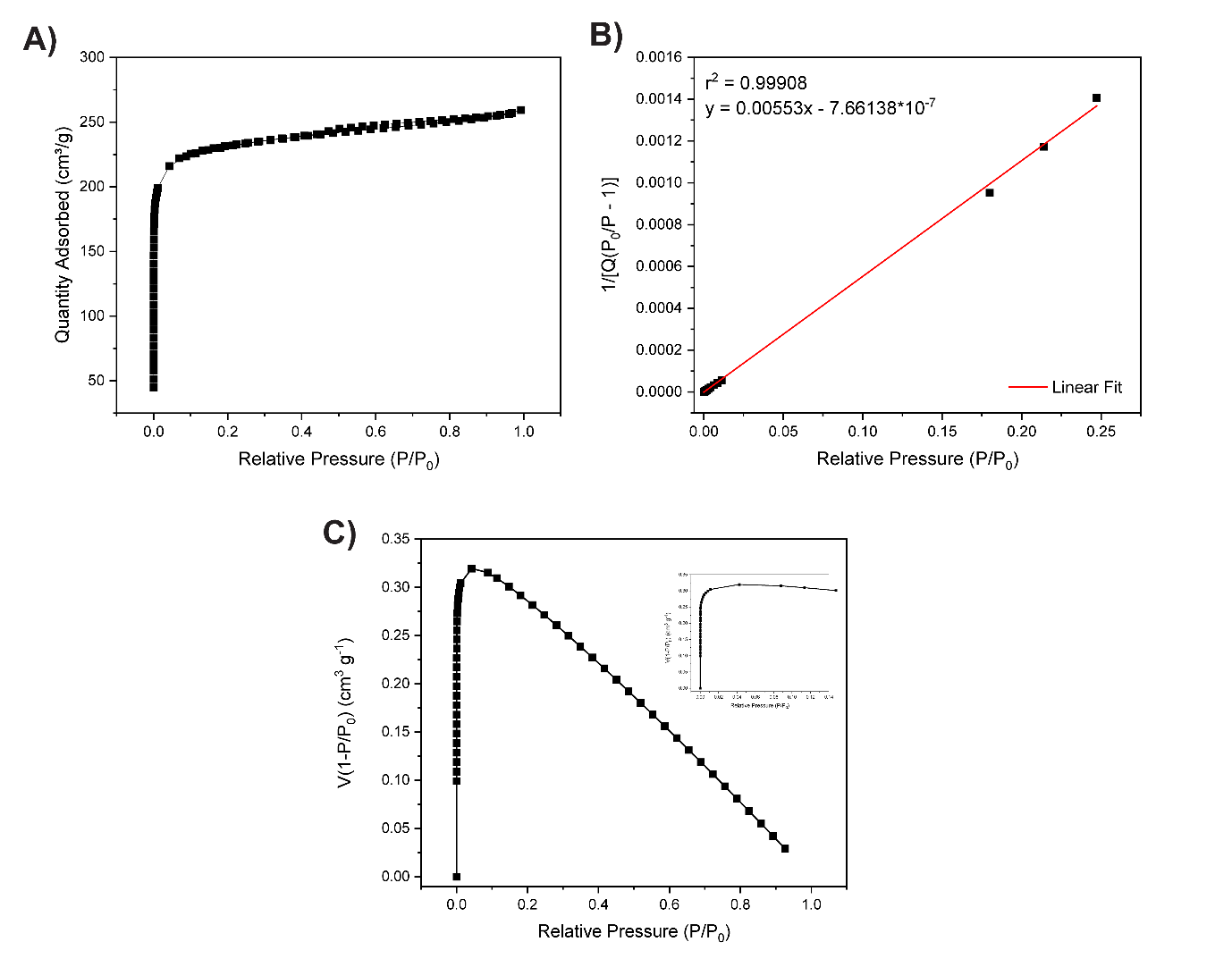


**Figure S18**. Textural properties of activated carbon: (A) BET surface area plot, (B) BET linear plot, and (C) Rouquerol plot. Based on the BET linear and Rouquerol plots, the specific surface area was determined to be **833 m²/g**.


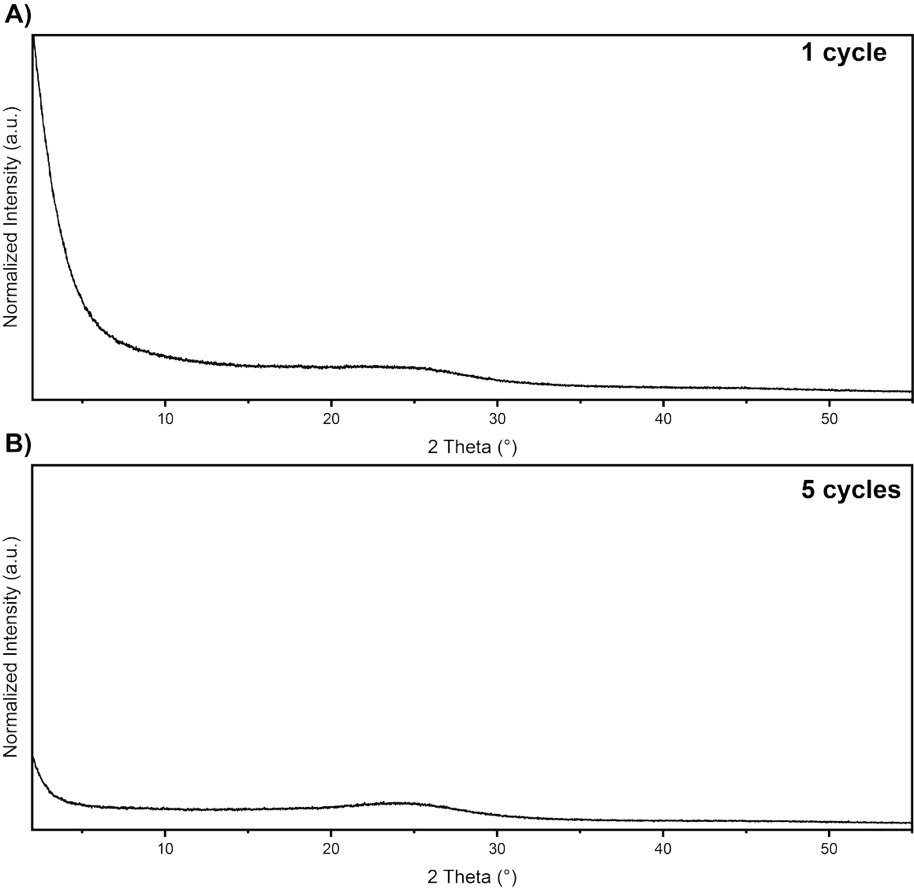


**Figure S19.** Powder X-ray diffractograms of PpTOC-350 after one cycle (A) and five cycles (B) of uptake and material recovery. Notably, no diffraction from Au nanoparticles can be observed.

**
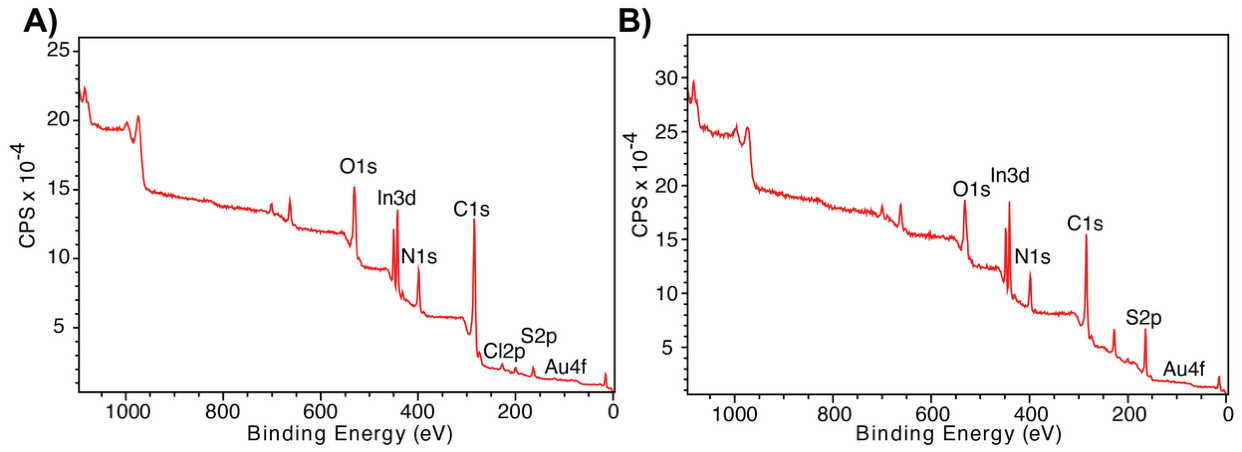
**

**Figure S20.** Survey spectra of PpTOC-350 after one (A) and after five (B) cycles of uptake and material recovery. Notably, the Au4f region is vacant; however, residual sulfur remains from the wash.

**
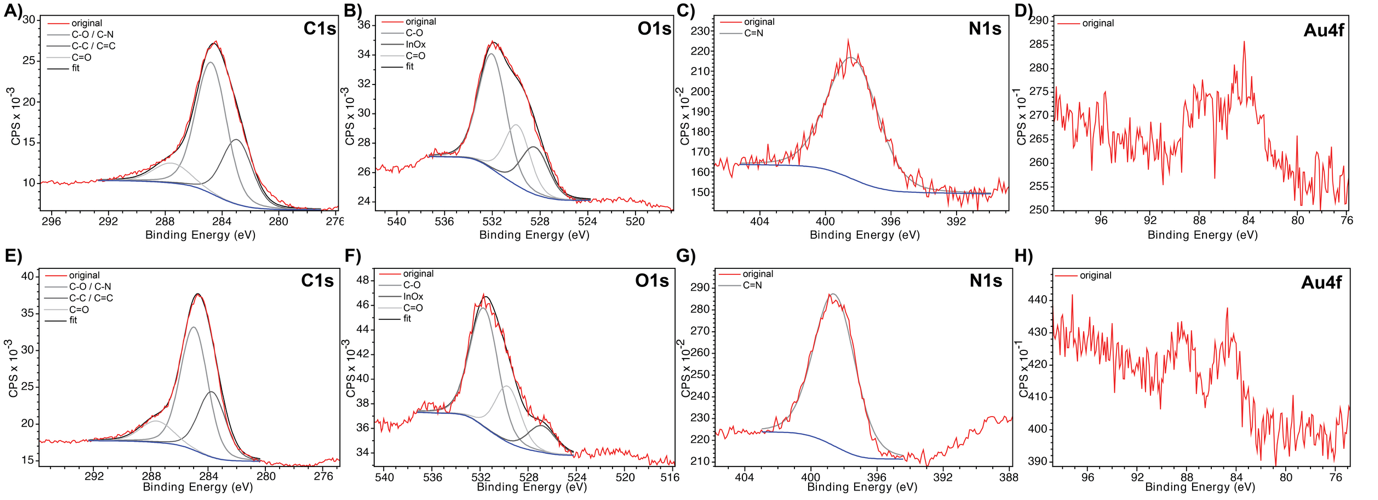
**

**Figure S21.** Deconvolutions of XPS narrow scans of PpTOC-350 after one (A – D) and after five (E – H) cycles of uptake and recovery experiments.


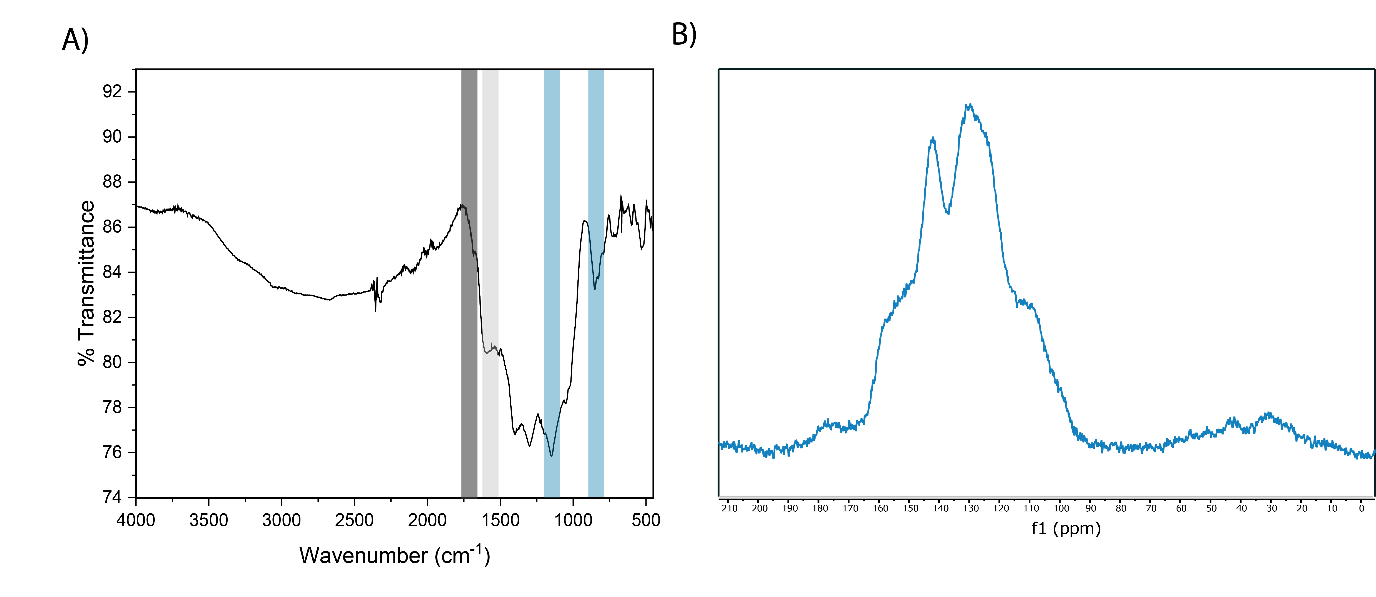


**Figure S22.** Fourier-transform infrared spectra of PpTOCs (A) and CP-MAS ^13^C NMR spectrum of PpTOC-350 (B) after recycling experiments.


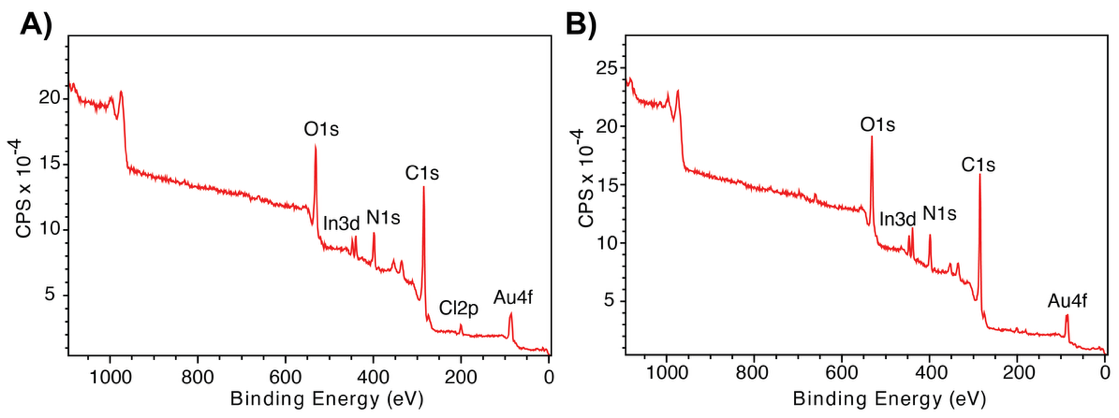


**Figure S23.** Survey spectra of PpTOC-350 before (A) and after (B) use in a catalytic experiment.


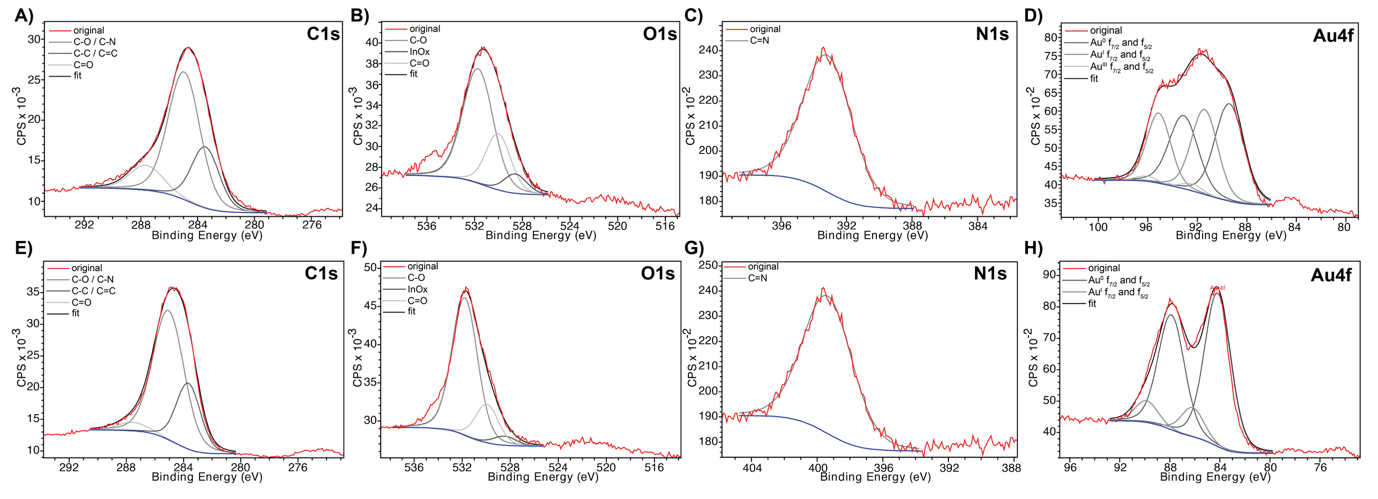


**Figure S24.** Deconvolutions of XPS narrow scans of PpTOC-350 before (A – D) and after (E – H) being used in a catalytic experiment. In the O1s deconvolutions, InOx refers to Indium oxides, which are present due to how the samples were prepared.

**
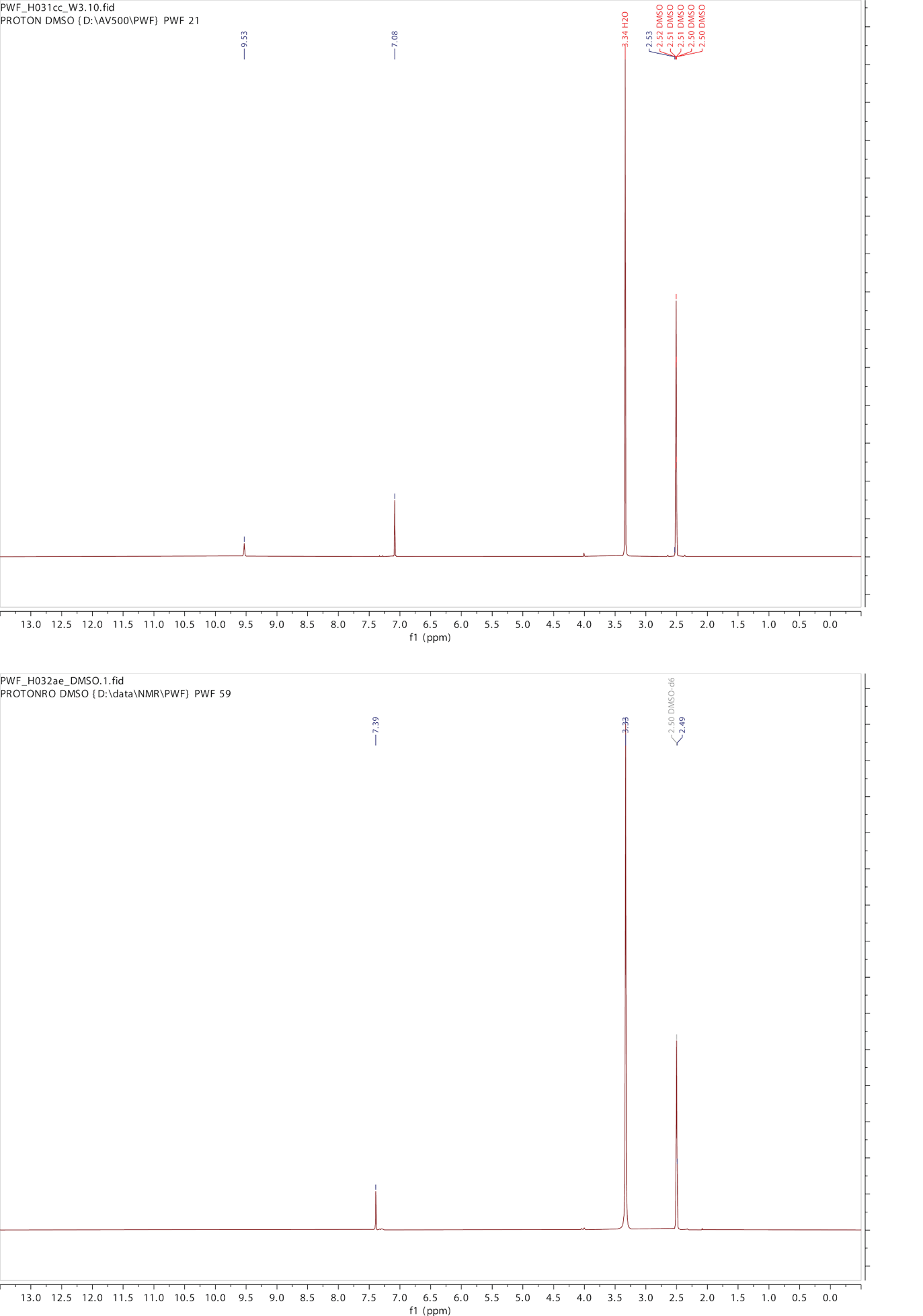
**

**Figure S25**. ^1^H NMR of tetrahydroxy phenazine (**4**, top, DMSO-*d_6_* 500 MHz) and phenazine tetrone (**5**, bottom, DMSO-*d_6_* 400 MHz).

**
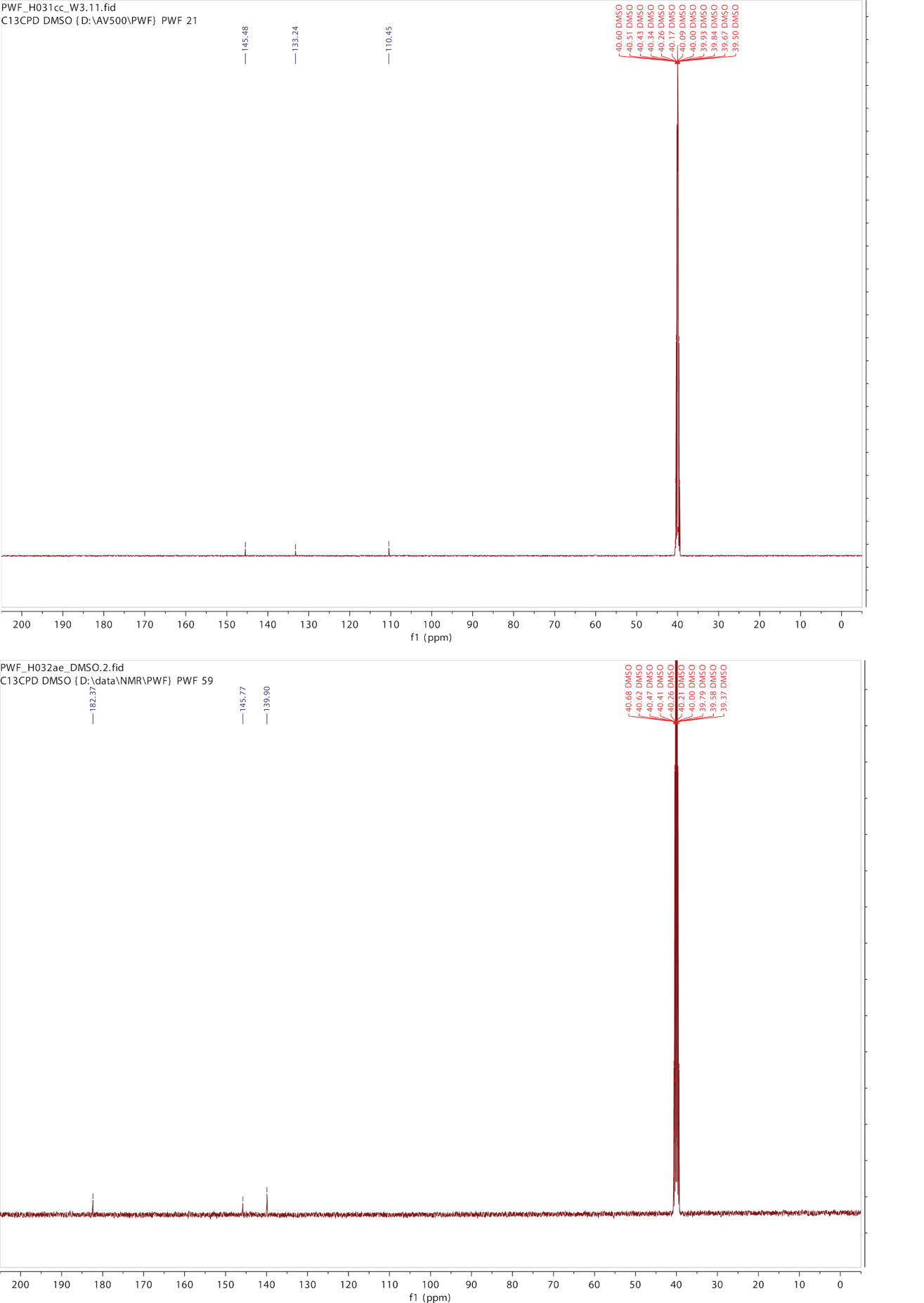
**

**Figure S26**. ^13^C NMR of tetrahydroxy phenazine (**4**, top, DMSO-*d_6_* 125 MHz) and phenazine tetrone (**5**, bottom, DMSO-*d_6_* 100 MHz).

**
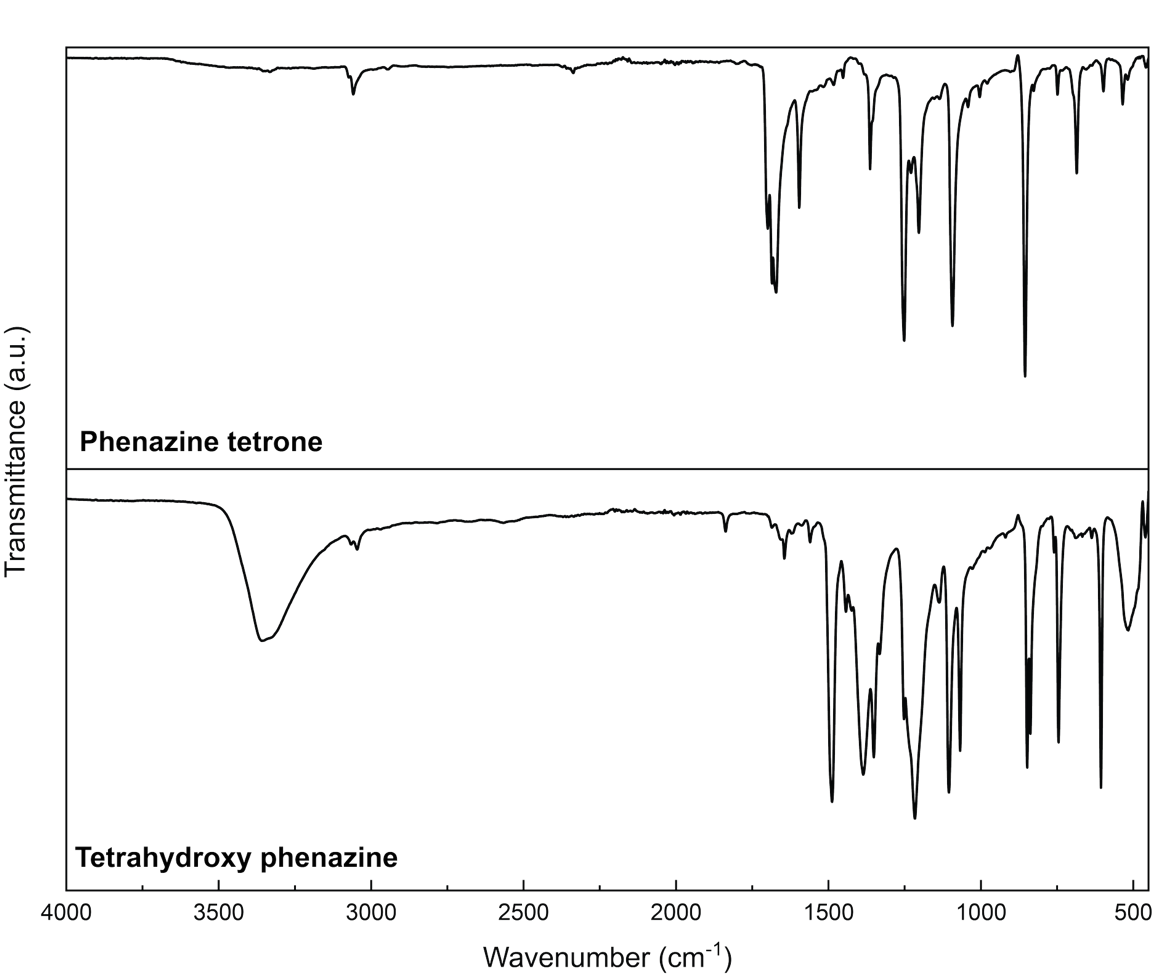
**

**Figure S27**. FTIR analysis of tetrahydroxy phenazine (**4**, bottom), and phenazine tetrone (**5**, top). The conversion of OH moieties around 3300 cm^-1^ to carbonyl moieties at 1690 cm^-1^ is clearly visible.


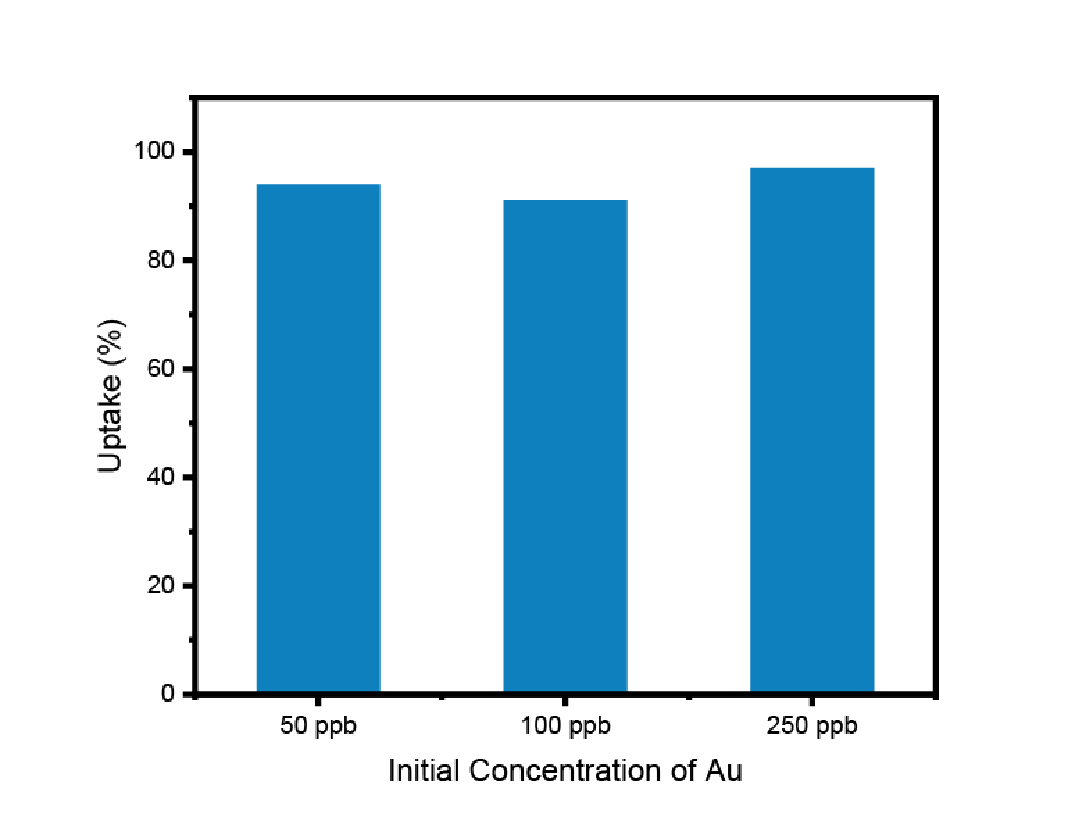


**Figure S28.** Gold uptake performance of PpTOC for ppb level of gold.

**Author contributions:**

AC, PWF, EA, and TA conceived, designed, and conducted the experiments. PWF, EA, and TA performed the material synthesis. PWF, EA, and TA characterized and analyzed the materials at different stages. KP performed NMR analyses. AC acquired funding for the project. PWF, AC, and EA co-wrote the manuscript.
